# Regulatory network-based model to simulate the biochemical regulation of chondrocytes in healthy and osteoarthritic environments

**DOI:** 10.1038/s41598-022-07776-2

**Published:** 2022-03-09

**Authors:** Maria Segarra-Queralt, Michael Neidlin, Laura Tio, Jordi Monfort, Joan Carles Monllau, Miguel Á. González Ballester, Leonidas G. Alexopoulos, Gemma Piella, Jérôme Noailly

**Affiliations:** 1 BCN MedTech, Universitat Pompeu Fabra, Barcelona, Spain; 2 Department of Mechanical Engineering, National Technical University of Athens, Athens, Greece; 3 IMIM, Barcelona, Spain; 4 Rheumatology Department, Hospital del Mar, Barcelona, Spain; 5 Orthopedic Surgery and Traumatology, Department, Hospital del Mar, Barcelona, Spain; 6 ICREA, Barcelona, Spain

**Keywords:** Biochemistry, Computational biology and bioinformatics, Systems biology

## Abstract

In osteoarthritis (OA), chondrocyte metabolism dysregulation increases relative catabolic activity, which leads to cartilage degradation. To enable the semiquantitative interpretation of the intricate mechanisms of OA progression, we propose a network-based model at the chondrocyte level that incorporates the complex ways in which inflammatory factors affect structural protein and protease expression and nociceptive signals. Understanding such interactions will leverage the identification of new potential therapeutic targets that could improve current pharmacological treatments. Our computational model arises from a combination of knowledge-based and data-driven approaches that includes in-depth analyses of evidence reported in the specialized literature and targeted network enrichment. We achieved a mechanistic network of molecular interactions that represent both biosynthetic, inflammatory and degradative chondrocyte activity. The network is calibrated against experimental data through a genetic algorithm, and 81% of the responses tested have a normalized root squared error lower than 0.15. The model captures chondrocyte-reported behaviors with 95% accuracy, and it correctly predicts the main outcomes of OA treatment based on blood-derived biologics. The proposed methodology allows us to model an optimal regulatory network that controls chondrocyte metabolism based on measurable soluble molecules. Further research should target the incorporation of mechanical signals.

## Introduction

Osteoarthritis (OA) appears from a combination of risk factors, among which aging and obesity are the most important. It affects approximately 22% of men and 31% of women over 55 years of age in 2019 according to the Institute for Health Metrics and Evaluation, provoking substantial morbidity and diminished quality of life^1,2^. The main hallmarks of OA are the degradation of articular cartilage and inflammation, which can cause intense pain and disability.

Several guidelines for OA treatment have been developed, mainly targeting pain reduction and functional restoration. They include conservative interventions, mostly based on painkillers, but total knee arthroplasty might eventually be required^3,4^. Disease-modifying drugs are not yet available because the mechanisms that alter homeostasis and pathophysiology of the articular cartilage involve complex and poorly understood interactions among genetic, biochemical, and mechanical factors^5^. Consequently, no interventions to repair degraded cartilage or decelerate the progression of OA are currently available^6^.

**Figure 1:**
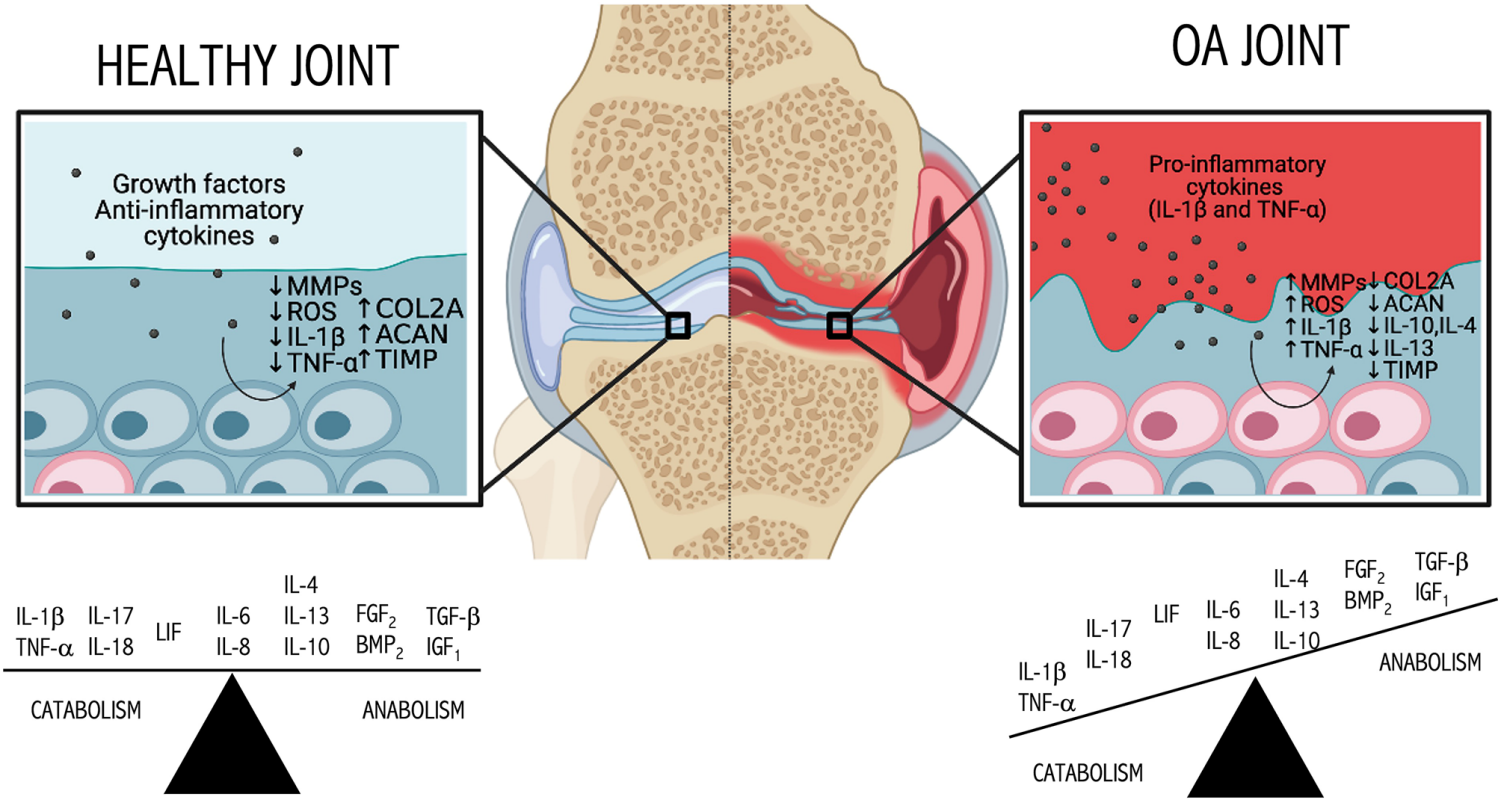
Involvement of regulatory proteins in chondrocyte and articular cartilage metabolism and homeostasis. In a healthy state, the predominant proteins are those that have a pro-anabolic nature, whereas for different reasons, when OA starts, pro-inflammatory cytokines increase and disrupt the balance between catabolic and anabolic molecules. In healthy articular cartilage (AC), chondrocytes remain in a quiescent state with very low turnover, and a balance between anabolic and catabolic cell processes is reached. In contrast, in OA, chondrocytes become dysregulated and start proliferating, increasing the production of inflammatory mediators and matrix-degrading enzymes, which causes mechanical weakness. Then, the basal activity of chondrocytes shifts into a more catabolic phenotype, and articular cartilage degrades as chondrocytes lose their ability to repair any damage in the surrounding extracellular matrix. Created with BioRender.com (2021).

Despite the multietiology of OA, it is suggested that secreted inflammatory mediators are critical players in OA pathophysiology, at least in early idiopathic OA^7^ (see Fig. 1). To better understand how chondrocytes stop working adequately to ensure the maintenance of joint cartilage, we should first apprehend the key biochemical interactions that control the balance between catabolic and anabolic processes.

Multiple in vivo or in vitro experiments have been performed to analyze the role of inflammatory mediators in the context of OA (see Additional Material 1A1 Table 1 in the Additional Material 1 with referenced bibliography). Nevertheless, researchers have not reached a clear consensus about any systematic chains of molecular mechanisms that explain chondrocyte dysregulation, and the complex interplay among the involved signaling pathways makes the challenge even larger. In this context, computational systems biology models offer integrative frameworks to analyze the interactions among complex sets of proteins, and directed protein–protein interaction networks/graphs that map the interactions among key cell regulators are particularly appealing. In such networks, the nodes are molecules (in the current study, mainly secreted proteins), and the edges or links represent the relationships among nodes and have specific directions. One of the main challenges is to integrate the most relevant nodes and acquire a proper network topology that might help understand how chondrocytes process signals, generate hypotheses and test causative cell responses to microenvironmental simulation^8,9^.

Soluble molecules, i.e. cytokines and chemokines, act as high-level cellular regulators of chondrocyte metabolism. The interactions between these proteins can be modeled as a network in the form of a continuous dynamical system. Dynamic models predict how a static protein-protein network may behave according to different initial nodal perturbations that may represent different measurable cartilage microenvironments through the analysis of the steady states (SS). In a dynamical system, SS are an example of an attractor that can be achieved with a given set of initial conditions. In cell biology, we compare the attractors or SS with long-term cell activity or phenotypes^9,10^.

There are different approaches to designing a static directed graph (i.e., a set of nodes connected by one-way edges or links): knowledge- or data-driven^11^. We hereby propose a combination of both approaches. When designing the knowledge-driven network, we did not consider intermediate metabolites. We assumed that such high-level modeling would allow calibration and direct comparison against experimental measurements of the chondrocyte secretome (data-driven approach)^12,13^. Eventually, we validated the ability of the model to predict semi-quantitatively reasonable changes in chondrocyte activity depending on the cell biochemical environment.

## Results

An initial prior knowledge network model was built by manual biocuration in the form of a literature-based protein interactome specific to articular cartilage and chondrocytes and, to some extent, to OA. Anabolic and catabolic generic interactions were further included by enriching the interactome with the protein–protein interaction database STRING^14^. The enriched network was then calibrated against experimental data through a genetic algorithm, leading to a new optimized network. Genetic algorithm is an optimization method that mimics the Darwinian theory of evolution. Based on an objective function, the algorithm chooses the best individuals (the most adapted) within each generation until the optimized solution is found (when a stopping criterion, defined by the user, is fulfilled). Each of the three protein interactomes was tested qualitatively and quantitatively by perturbating the model, according to independent experiments (quantitative test) and literature knowledge (qualitative test). For the qualitative test, we checked whether the model could replicate well-established chondrocyte behaviors in OA (see Additional Material 1A1 Table 2 in the Additional Material). An extensive quantitative assessment was performed by computing (i) the normalized mean absolute deviation (NMAD) to measure the cumulative error between the predicted and the experimental datasets, and (ii) the normalized root squared error (NRSE) for each simulated data. To sum up, a 3-step methodology (reading-enriching-optimizing) is developed to establish a first immunomodulatory network-based model that can control chondrocyte metabolism under different biochemical environments, in a context of early idiopathic OA.

### Literature based network (LIT) model

**Figure 2:**
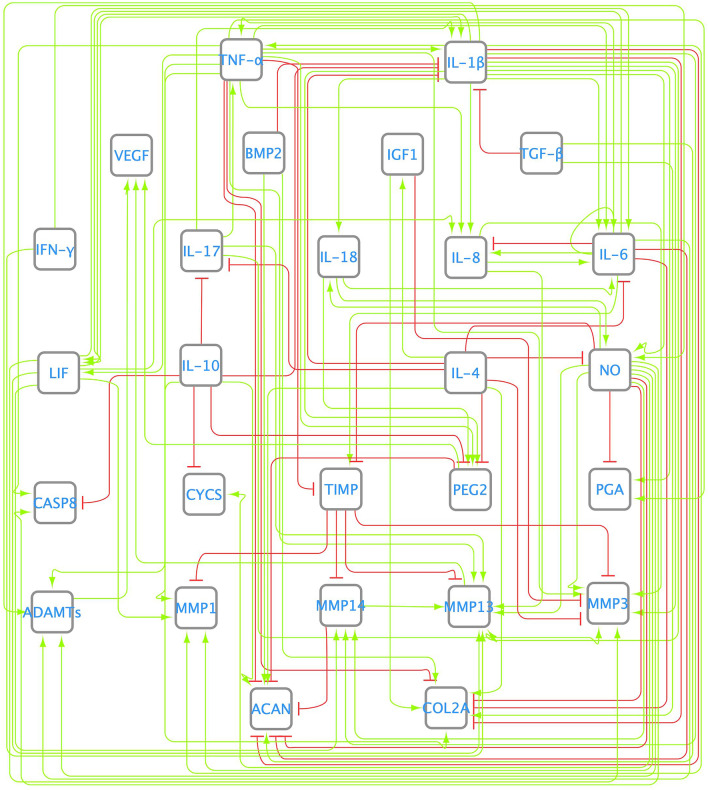
Literature-based network (LIT). Nodes of the network correspond to the most relevant osteoarthritis-related molecules. Nodes are interconnected with each other with a set of activating (green) and inhibiting (red) links or edges. The summarized connectivity can be seen in Additional Material 1A1 Table 3.

**Figure 3:**
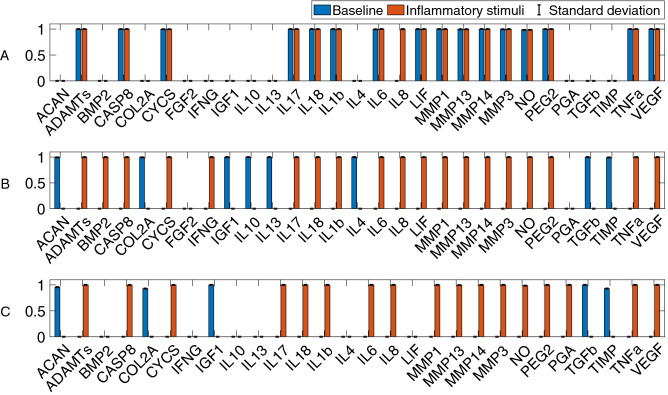
Final steady states of the baseline (blue) and proinflammatory stimulations (orange). The three networks developed are represented: (**A**) the literature-based network; (**B**) the enriched network; and (**C**) the optimized network.

The conducted search allowed us to select a total of 63 articles. Data from these works were used to design the literature-based network (LIT) depicted in Fig. 2. Figure 3 A (blue bars) shows that the basal steady states (SS) of the network converged to a catabolic state: proinflammatory-related nodes and degrading enzymes have a final expression close to 1. The LIT network stimulation with the main proinflammatory cytokines involved in OA (IL-1β, TNF-α, IL-6, IL-8, IL-17 and IL-18) led to a catabolic-like SS. Therefore, no significant changes were observed between them (t-test, α = 0.05). Qualitative evaluation further shows that the LIT network can only predict 75% of the reported behaviors summarized in the additional material section Additional Material A1 Table 2. Finally, this network yields an absolute mean deviation (AMD) value of 0.1660 (i.e., approximately 16.5155% of cumulative error expressed as normalized mean absolute deviation, NMAD) when used to reproduce the experiments by the Melas et al.^12^ experimental data set. When calculated against Neidlin et al.^13^, the cumulative error was 6.5412%.

### Enriched network (ENR) model

**Figure 4:**
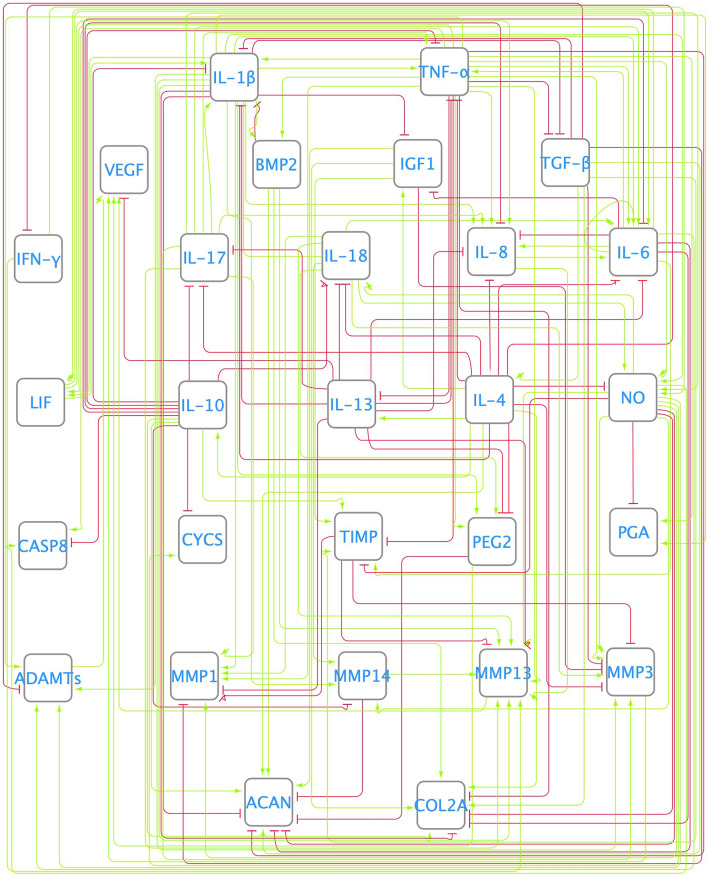
Enriched network (ENR). It was performed with the help of a protein–protein public database STRING^21^. The summarized connectivity can be seen in Additional Material 1A1 Table 3.

Network enrichment with high-throughput tools allowed us to introduce interactions never reported before in chondrocytes, as well as missing links not found manually in the literature-based network model (Fig. 2), such as the inhibition of TGF-β by IL-1β and TNF-α (see Additional Material A1 Table 3 in the additional material section). In Fig. 4 it can be seen the enriched directed graph. The t-test showed that enrichment led to significant differences between the basal and perturbed SS after proinflammatory node stimulation (Fig. 3 B (orange bars)). In the basal state, nodes corresponding to anti-inflammatory cytokines (interleukin 10 (IL-10), interleukin 13 (IL-13) and interleukin (IL-4)), structural proteins (COL2A and ACAN) and TIMP were activated. In contrast, after proinflammatory stimulation, degrading enzymes and nociceptive cytokines (PEG2 and VEGF) reached maximum expression, among other catabolic nodes. Furthermore, COL2A and ACAN became inhibited, as well as growth factors such as IGF_1_ and TFG-β. Qualitative evaluation showed that the ENR model predicted 57% of the reported chondrocyte metabolic characteristics well. The value of objective function when compared to the Melas et al.^12^ data (training set) is 0.0812 (approximately 13% in terms of cumulative error). When computed against Neidlin et al.^13^ (test data set), the cumulative error was 10.6024% expressed as a normalized mean absolute deviation (NMAD) value.

### Optimized network (OPT) model

**Figure 5:**
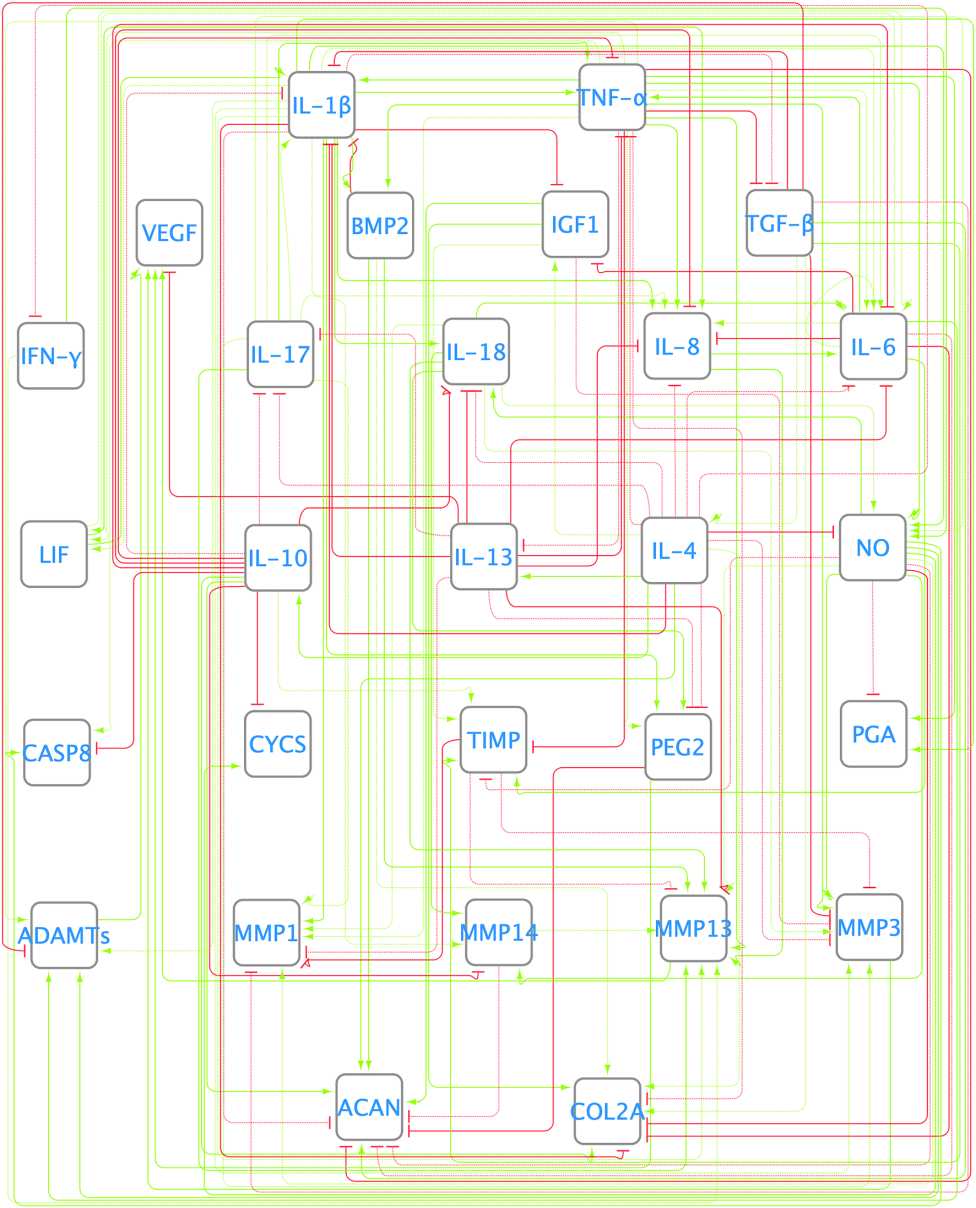
Optimized network (OPT). It was achieved by an optimization process based on a genetic algorithm. The dotted lines are the ones that the GA has eliminated. The summarized interactions can be found in Additional Material 1A1 Table 4.

**Figure 6:**
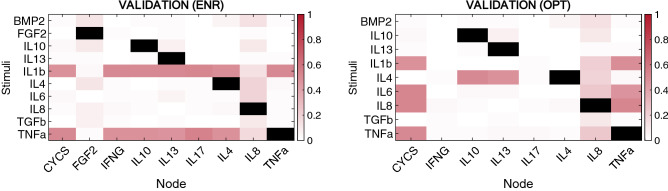
Normalized root squared error (NRSE) data point by data point before (at left) and after (at right) the optimization for the test set. Black squares are not considered when calculating the NRSE because the same protein used to perturb the model was then measured.

After topology optimization (see directed graph in Fig. 5), the normalized mean absolute deviation (NMAD) further decreased from around 13 to 12%. The optimized network (OPT) could integrate well pro-inflammatory stimuli that lead to a significant change in the steady state. Figure 3 C shows that when a catabolic stimulus (orange bars) was applied, the network went from a healthy state to a catabolic steady state: degrading enzymes and proinflammatory cytokines (IL-1β, IL-17 or IL-6) had high expression rates, and anabolic factors were no longer expressed (structural proteins and almost every growth factor). Independent validation calculations revealed that OPT had a higher capacity than the ENR to reproduce independent experimental data by Neidlin et al.^13^ from 10.6024 to 7.9420% of NMAD. Moreover, qualitative evaluation showed that the optimized network could reproduce 95% of the expected chondrocyte activity, as reported in the literature. Figure 6 shows the decrease in the Normalized Root Mean Squared Error (NRSE) for each data point before (on the left) and after (on the right) the optimization step. Then, in Fig. 6, it can be seen NRSE for the validation data set, with an 81% accuracy in the OPT model.

### Proof of concept

We replicated Frizziero et al.^14^ treatment with our OPT model with the three different initial conditions described in the Methods. The t-test (α = 0.05) indicated whether the simulated scenarios significantly changed the catabolic steady states (SS) of the OPT model to a more anabolic SS.

**Figure 7:**
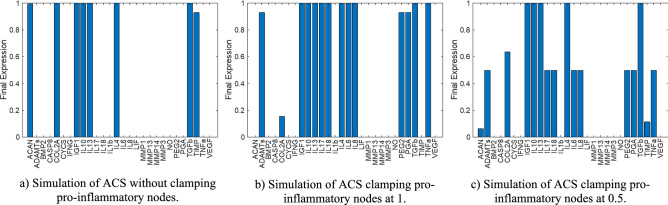
Proof of Concept steady states for each scenario. (**A**) First scenario: optimized network simulated with autologous conditioned serum (ACS). (**B**) Second scenario: Proinflammatory nodes of the optimized stimulation at their maximum capacity along with ACS stimulation to simulate an osteoarthritic joint or environment. (**C**) Third condition: proinflammatory nodes of the optimized stimulation at half of their maximum capacity along with ACS stimulation to simulate an osteoarthritic joint or environment with partial modulation of proinflammatory cytokines.

- Scenario 1: Autologous conditioned serum (ACS) molecules clamped at 1. Figure 7A shows that catabolic nodes and pain-related nodes (PEG_2_ and VEGF) reduced their expression, and pro-anabolic and structural proteins recovered with significant differences compared to the initial catabolic SS.
- Scenario 2: Both proinflammatory cytokines and anabolic factors from ACS clamped at 1. Figure 7B shows that MMPs and pain-related molecules were reduced, but the expression of structural proteins was not restored. No significant differences with catabolic SS could be appreciated according to the t-test.
- Scenario 3: Anabolic nodes from the ACS clamped at 1; catabolic cytokines clamped at 50% of their potential. Figure 7I shows that the perturbed model could restore the levels of COL2A but not ACAN. MMPs and pain-associated nodes (VEGF and PEG2) were reduced. Significant differences were calculated compared to the initial catabolic SS.

## Discussion

In osteoarthritis (OA), cellular and matrix changes occur because biochemical stimuli alter chondrocyte function, eventually leading to articular cartilage composition and architecture changes. Experimental measurements provide a wealth of information about the influence of single anti- or proinflammatory mediators or growth factors on chondrocyte protein expression. To date, the comprehensive integration of such information governing OA development remains limited^15^. Systems modeling approaches can, however, complement experimental work, and computational models have been widely used in the fields of cancer, cardiovascular diseases, and neurodegeneration but are not so widely exploited in OA research^16^.

In this study, a new in silico network model was developed to represent biochemical interactions at the cell level, and it successfully replicated known chondrocyte behaviors in healthy and OA environments. Our computational model is interpretable in the sense that it represents current biological knowledge about chondrocyte immunogenicity in the context of early idiopathic OA. Although our model can predict early molecular markers of hypertrophy, it does not target advanced events of OA progression, such as the switch from healthy to hypertrophic chondrocyte, or fibrotic remodeling of cartilage^17^.

Several examples in the literature further corroborate the potential of computational systems biology models and simulations to support the combat against knee OA: Proctor et al.^16^ developed a computational model of the intracellular regulatory network that controls the synthesis/degradation of collagen by chondrocytes; Hui et al.^18^ exploited the impact of age-related molecules on the development of OA; and Kerkhofs et al.^19^ studied the dynamic behavior of a regulatory network that governs chondrocyte hypertrophy from a resting chondrocyte. However, current regulatory networks reported for chondrocytes do not exactly target the effects of cytokine dynamics in the onset of idiopathic OA. Rather, they focus on intracellular regulation details. To our knowledge, our regulatory network is the first to integrate the respective effects of different microenvironmental cell signals on effective cell activities, admitting soluble cytokine information as model inputs. Simulations captured the expected normalized influence of different regimens on the capacity of chondrocytes to retain full anabolic activity or provoke extracellular matrix degradation.

We show hereby that high-level semiquantitative regulatory networks can be built and summarize the molecular regulation of articular chondrocytes in a variety of measurable biochemical environments. The literature-based network model was catabolic by default, pointing out the bias possibly induced through a knowledge-driven modeling approach due to the large quantity of information related to catabolism compared to anabolism regarding cartilage biology and OA. Both semiquantitative and qualitative assessments of the simulations revealed that this shortcoming impaired the capacity to develop reliable knowledge-driven models.

However, we could successfully improve the literature-based network (LIT) model out of public databases. The enrichment used mining of both chondrocyte-specific and nonspecific literature and allowed the enriched network (ENR) model to reflect the biosynthetic activity of a normal chondrocyte in terms of aggrecans and type II collagen, in contrast to the LIT model. Anabolic information has never been reported before for chondrocytes, such as the activation of IL-10 and IL-13 by IL-4. These reactions are commonly associated with these cytokines, as this information derives from curated pathways. However, to the best of our knowledge, this cell type has never been stated before. In addition, we included OA interactions that were not identified through manual curation of the specific OA literature. For example, the inhibition of TGF-β by TNF-α and IL-1β seemed to be cornerstone for the anabolic-catabolic switch of the networks when a pro-catabolic environment was simulated. Interestingly, Roman-Blas et al. reported a similar behavior in bovine chondrocytes, highlighting the potential of the model to generate relevant information and hypothesis for the experimental exploration of the regulation of cartilage metabolism^20^. Actually, the optimization process eliminated the inhibition by IL-1β and preserved the inhibition by TNF-α, highlighting the key role of this molecule which is often pointed out as an important marker of low-grade inflammation. This suggests that anti-TNF-α treatments might be promising in terms of cartilage regeneration approaches, rather than the usually tested anti-IL-1β drugs.

Node-edge graphs are collected in several public databases and aim to represent the current knowledge of the scientific literature about the entire interactome (i.e., STRING^21^, KEGG^22^, REACTOME^23^). Despite an improvement of the enriched network (ENR) model compared to the literature-based (LIT) network, several limitations remain and are difficult to identify and control in network modelling. On the one hand, biases might still be present (e.g., the proteins supposed to be more relevant are better studied). On the other hand, ambivalent behaviors, i.e., conflicting opinions in the literature, can lead to problems in decision making from the perspective of the model developer. But, they can be used as an initial step to create a knowledge-derived network or to enrich the interactions manually retrieved from a set of specialized articles. However, it has biases. To overcome this, we fitted the enriched network (ENR) model with metabolomics data, which come from cytokine release measurements after stimulation of biological samples, through a genetic algorithm (GA) using absolute mean deviation (AMD) as a fitness function. We chose the AMD over other conventional fitness functions because it seemed to provide the GA with improved flexibility for the adjustment of node connectivity and led to the most robust solutions in terms of biological interpretability. Indeed, the AMD allowed us to achieve excellent qualitative and relatively good quantitative assessments against independent data. However, one must be cautious with error compensations due to possible opposite signs of nodal errors, e.g., potentially leading to extremely low error descriptors. Here, we used the normalized mean absolute deviation (NMAD), which reflected the absolute cumulative error over 67 individual node responses and led to a mean global error of 8% for the whole optimized network (OPT) model, which was deemed accepted in light of the qualitative assessment results. The Normalized Root Squared Error was calculated in addition to the NMAD to deeply examine the error cancelation effects in the OPT model, and 81% of the predicted responses had an error below 13%.

The improvement of the response of the data-driven optimized network (OPT) model compared to the enriched network (ENR) model reveals that knowledge-driven network approaches benefit from experimental data, possibly decreasing information bias. However, in data-driven approaches, it is difficult to infer biologically relevant outcomes beyond the influence of the reduced set of specific data available for quantitative calibration and validation. In fact, the literature-based network (LIT) network led to lower normalized mean absolute deviation (NMAD) values than the ENR and OPT models when evaluated against the independent data by Neidlin et al.^13^, but the qualitative validation of this network failed completely. For this reason, fluxomics is being increasingly used to enhance model interpretability in data-based metabolic modeling^24^. Provided such quantitative assessments are also considered, the results of our qualitative evaluation, with up to 95% accuracy and sound predictions of the outcome of autologous conditioned serum (ACS) treatment, support that the use of steady states information is helpful to develop biomedically relevant hypotheses for computational and systems biology models. A remarkable novelty is the prediction of biologically interpretable and contrastable steady states for articular chondrocyte metabolic activity under normal conditions and in a plurality of catabolic, anabolic and mixed biochemical environments based on measurable soluble factors. Our evaluation supports that our model can predict a wide range of chondrocyte response to biochemical perturbations, according to both knowledge (see qualitative evaluation in the “Results” section) and data (see quantitative validation in the “Results” section), independent of the network design and calibration processes. Besides, it is possible to include the possible regulation effects by the extracellular matrix (see Additional Material 3).

Regulation networks can be modeled with various mathematical approaches. The most commonly used models are Boolean and ordinary differential equation models. We are interested in the second one since it can describe transient and (semi) quantitative cell responses. However, the cost is a high number of reaction parameters that are difficult to define. Different methodologies are presented to extract dynamical behavior (i.e., Mendoza et al.^25^ or Krumised et al.^26^) from nonspecific information to translate discrete node-edge graphs into continuous systems. Dynamic analysis can simulate the temporal evolution of the different nodes under specific conditions and to study the possible outcomes (steady states, SS) of the established network. In fact, our optimized network (OPT) model reaches the appropriate SSs under pro- and anti-inflammatory cytokine stimulations, so it can properly integrate the most studied cytokines involved in OA: IL-1β perturbation in the OPT model can strongly activate MMP13 and decrease structural proteins (ACAN and COL2A). These biochemical events are major hallmarks of OA chondrocytes. Another interesting feature of our model is the multiple ability of NO to induce apoptosis-related proteins (CYCS and CASP8), IL-18 (a proinflammatory cytokine) and degrading enzymes, even though no current experimental data were available for ROS. Qualitative results show that the outcome of our OPT network following NO perturbation is in accordance with what is expected in chondrocytes^27,28^. NO in cartilage cells is closely related to increasing the expression of proinflammatory nodes, especially IL-18. Our OPT model mimicked this behavior, as it increased the expression rate of IL-18 after NO stimulation. This type of behavior, as far as we are concerned, has only been attributed to chondrocytes^27,29^. It is true that we have found several interactions concerning secretome chondrocyte information based on cytokine perturbations. However, not all them have been checked directly in chondrocytes without scanning the effect of signaling pathways. This approach has therefore highlighted the need for experimental data in this field.

There are other viable approaches to fit experimental data, such as CellNopt^30^, SigNetTrainer of the CellNetAnalyzer toolbox^31^, DoRoTHea^32^ or ANIMO^33^. We choose a genetic algorithm (GA) process over such tools for several reasons. First, CellNopt’s fitness function is based on Boolean logic models, and we were interested in continuous network models. Additionally, it does not ensure global error minimization, as we did by using the absolute mean deviation (AMD) in a GA. Second, CellNetAnalyzer was built for microorganism engineering purposes. DoRoThea was meant to predict transcription factor activities from the gene expression of their targets (we hereby focused on higher regulation processes). Finally, with ANIMO, it is possible to determine how fast one edge influences a node with respect to others, for intracellular networks (network kinetics). As we work with a high-level cell activity model, it is difficult to establish edge’s kinetics. In contrast, the methodology in Mendoza et al. ensures that reaction kinematics does not affect the steady states of the system through the distribution of α and β parameters [see Eqs. (6) and (7)], being the latter uniquely defined by the knowledge- and/or data-driven topology. Then, our own optimization process led to the prediction of basal activation of growth factors, i.e., TGF-β and IGF_1_, that are important for the anabolic activity of healthy chondrocytes and cartilage homeostasis^34,35^. The GA used for the optimization of the enriched network (ENR) model drastically reduced the AMD between experimental and predicted data and, as discussed before, led to a positive independent quantitative validation of the eventual optimized network (OPT) model when assessed against multiplexed protein measurements by Neidlin et al.^13^ (Fig. 6). On the one hand, our OPT model can reproduce experimental cytokine release data better than the ENR model when independently validated. On the other hand, independent validation showed that the training step did not generate any overfitting. The OPT model has the ability to predict a wide range of biochemical stimulations, which was confirmed by the 95% accuracy achieved during the qualitative evaluation against 69 expected cell behaviors (Additional Material A1 Table 2).

The selection of the final optimized network (OPT) model network required a trade-off between quantitative fit and interpretability. We explored 1800 networks with different objective functions and carefully analyzed the best networks according to experimental outcomes and/or literature-based behaviors. As shown in the Additional Material 2, the genetic algorithm (GA) often found a solution that had a low fitting error but lacked biological plausibility. As our model was based on knowledge, it is hard to think that the network is not working for the purpose for which it was designed, i.e., integrate the current knowledge in chondrocyte metabolism in a network-based model. We found networks that were able to represent almost every individual response in the dataset used for the independent quantitative validation (with 88% accuracy). However, these networks could only reproduce 50% of the knowledge-based responses, with some of the predictions that were simply nonsense, biologically speaking. In contrast, we also found networks with excellent qualitative evaluation (at least 90% accuracy), but they were catabolic per se and/or led to poor quantitative independent validation and/or were unable to replicate autologous conditioned serum (ACS) treatment outcomes. Our selected OPT model offered excellent interpretability (it could represent 95% of the knowledge-based responses tested) with a notorious ability to represent experimental responses, and 81% of the responses quantitatively tested had an error lower than 13%. Remarkably, the quantitative simulation-experimental mismatches could be explained in terms of biological features. For example, high errors involving the CYCS node are likely due to the intracellular nature of the protein^36^, whereas we were working mainly with secreted proteins. Second, the network response in terms of IL-13 and IL-10 activation upon IL-4 stimulation can be associated with the lack of mechanotransduction pathways, which is further discussed below.

Controversially, in the baseline of our optimized network (OPT) model, the expression rate of IL-4 was 0, i.e., the same as in a pro-inflammatory situation, whereas several studies report that IL-4 is synthesized by healthy chondrocytes, and expression is reduced when OA appears^37^. Our model could not capture these changes in IL-4 expression. One possible explanation is that the generation of the model targeted information strictly limited to biochemical cell regulation, whereas IL-4 expression in normal chondrocytes is strongly associated with mechanotransduction pathways. Additionally, in the validation set by Neidlin et al.^13^, healthy cartilage explants were cultured under static conditions, and IL-4 production was not significant. Arguably, the authors also simulated OA cartilage explants through biochemical degradation of the healthy samples, and the production of IL-4 was activated by IL-1β stimulation. However, because enzymatic degradation modifies the turgence of the tissue structure, it is not clear whether mechanotranduction would not have initiated such a response. The experiments from Sadler et al.^38,39^ demonstrated the relationship between integrin signaling and the release of IL-4 when chondrocytes were exposed to mechanical pressure^40^. IL-4 is a major active autocrine/paracrine signaling molecule able to induce an anabolic response of normal chondrocytes, which appears to be impaired in OA cartilage^37^. Accordingly, when our optimized model is initialized with the IL-4 node set to its maximum expression (i.e., 1), proanabolic (IGF_1_, TGF-β, TIMP), anti-inflammatory (IL-13, IL-10), and tissue structural nodes (COL2A and ACAN) involved in cartilage preservation achieve their maximum activation. However, the development of new models should explore the possible primary regulation of IL-4 and its effects through specific mechanotransduction pathways.

To see how our model could replicate an  in vivo independent situation for chondrocytes, we reproduced a treatment explained by Frizziero et al.^14^ based on autologous conditioned serum (ACS). Coherent results were obtained even though it is difficult to know exactly which should be the effect of the treatment on the initial conditions of the model in terms of inflammation, i.e., conditions largely controlled by the synovium, a priori. Therefore, we propose three different situations.

- The first scenario, with no stimulation of pro-catabolic nodes, could completely repair the protein expression profile of healthy chondrocytes with significant differences. No inflammatory mediators (principally TNF-α, since IL-1β was blocked) were expressed, but pain-associated molecules (PEG_2_ and VEGF) were expressed, and structural proteins were re-expressed, suggesting that ACS might have the capacity to restore degraded cartilage. However, according to Frizziero et al.^14^, no restoration of the articular cartilage could be seen. Hence, assuming that pro-catabolic nodes would simply shut down is probably too optimistic, since it suggests that synovitis, low-grade inflammation or the effect of undue mechanical loads on degraded cartilage would be fully stopped.
- The second scenario, when trying to simulate a more catabolic environment (as expected in an OA situation), was consistent with the reported treatment by Frizziero et al.^14^: our model suggested that VEGF and PEG_2_ were not re-expressed (i.e., pain reduction) and that no recovery of structural proteins can be observed (neither ACAN nor COL2A were re-expressed), which explains the lack of cartilage restoration observed clinically. However, the differences between the compared steady states of the model were not statistically relevant.
- The third scenario, with proinflammatory nodes clamped at 50% of its potential, showed a significant difference between steady states. In this case, COL2A was recovered, but not ACAN. Inflammatory nodes in the network had higher expression rates, but the inhibitory inputs from ACS reduced both the expression of MMPs and the activation of pain-associated nodes. In addition, when IL-1β had no restrictions, COL2A had higher expression rates. These results were consistent with the reported results of the treatment in vivo: pain was reduced, but the treatment failed to repair the damage from ECM.

Overall, the proof of concept shows that the outcome of the system (in terms of the expression of the nodes) depends on the initial states of the nodes. The mathematical model, generated by combining previous knowledge and specific experimental data, is able to replicate independent sets of both experimental data and literature-based behaviors, beyond the specific data used for the development. Furthermore, the predicted molecule regulations can qualitatively explain the outcome of a clinical treatment based on biologics. The results of our case study simulations also indicate that anti-IL1β therapies might not be sufficient to cope with OA in clinical trials, as highlighted in the literature^41^. And might be focused on anti TNF-α therapies, as the optimization process suggests. From a methodological point of view, this outcome reveals the importance of the baseline for network-based models. Furthermore, given the nature of its nodes (i.e., soluble extracellular molecules), our network can easily be patient-specifically initialized with data from synovial fluid analyses.

Our model is designed to explore early idiopathic OA through cytokine stimulation. Such framework shall also be useful to explore the effect of metabolic syndromes on the triggering of OA, as those syndromes are also related to the release of pro-catabolic cytokines in the synovium. Metabolism-specific nodes such as lipopolysaccharide, leptin and adiponectin would nicely complement the capacity to target metabolic origins of OA as they have been identified with inflammation^42^. Yet, as discussed before, a first priority is to complement the model by adding mechanotransduction pathways and combine the latter with the current cytokine signaling, which would also pave the way to explore traumatic origins of OA (secondary OA). In the meantime, the current model to describe the influence of immunogenicity in early OA is cornerstone for these developments towards the prediction of third causes of OA.

Further developments should tackle the dynamic stimulation of chondrocytes cytokine diffusion from the synovium, bone and the possible communication among chondrocytes (cell–cell signaling) at tissue level in longer terms through an agent-based model. But how autocrine and endocrine signaling might affect chondrocyte metabolism will be dictated by this network modelling approach, as it can integrate the action of soluble factors no matter its source tissue. Besides, in this work, we used the data in Neidlin et al. for validation, which involves samples from only two patients. In these experiments, no batch effects due to patient-to-patient variability were observed, and all experiments proved to be repeatable (quadruple tests). Hence, while the data are deemed of good quality, including more data from different patients shall support future developments of statistical modelling to address the important question of uncertainty associated to inter-subject variability.

In conclusion, an interpretable network-based model able to explain nontrivial interactions among the main biochemical factors involved in early idiopathic OA was developed, which eventually leads to specific regulation of articular cartilage structural proteins. But, this cell-level model cannot readily capture changes at tissue level, which would require a description of the communication both among chondrocytes and adjacent cartilage tissues. Indeed, it could stand the basic rules of a chondrocyte behavior (when used as an agent) in an agent-based solver to control tissue volume responses. However, our model is the first, to our knowledge, to readily incorporate soluble cytokines that are directly measurable in the synovium. Then, it might be a powerful tool to better understand and anticipate the effect of OA-modifying drugs.

## Methodology

### Overview

**Figure 8:**
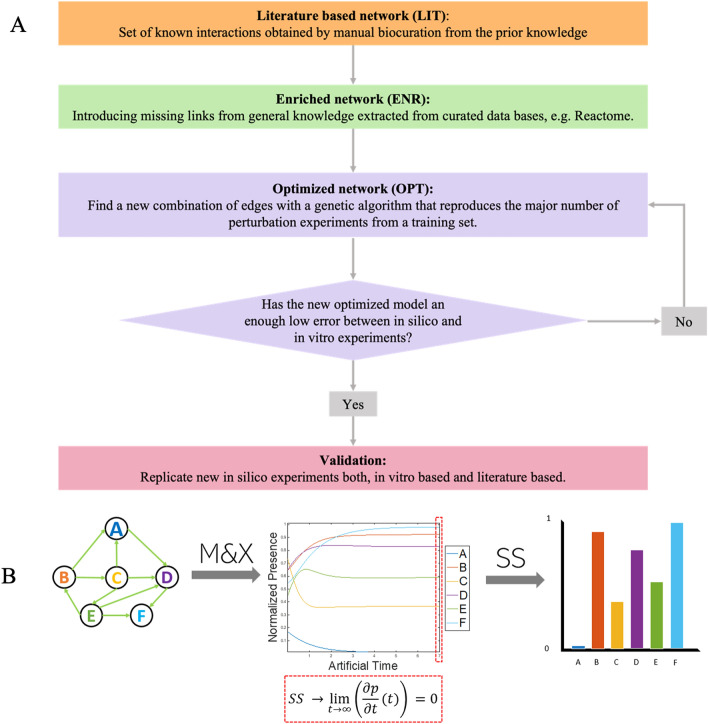
Summarized methodology. (**A**) Workflow for the network design. The literature-based (LIT) network was built from available biological knowledge from specialized journals. Then, STRING^21^ was used to complement interactions, obtaining the enriched (ENR) network. Finally, a genetic algorithm (GA) and experimental data were used to tune the node interactions, leading to the optimized (OPT) network. (**B**) Illustration of how the steady state of the system is extracted from the dynamic information provided by the network calculations. (M&X is an acronym for the Mendoza et al.^25^ methodology).

The first interactome was built from a corpus of peer reviewed articles. As the results suggested that the network was catabolically deviated, STRING^21^ was used to enrich the network with interactions from general knowledge^21^ leading to the enriched network (ENR) model. Finally, the ENR network was calibrated against experimental data with a genetic algorithm (GA); the resulting network was called the optimized network (OPT) model (see Fig. 8A). Then, network was validated against experimental and knowledge based data.Each of the three protein interactomes (LIT, ENR, OPT) was tested qualitatively and quantitatively. For the qualitative test, we checked whether the model could replicate well-established chondrocyte behaviors in OA (Additional Material 1A1 Table 2). An extensive quantitative assessment was performed by computing the normalized mean absolute deviation (NMAD)

1 $$NMAD=\frac{\sum_{n}^{N}\left|\frac{{x}_{n}-{y}_{n}}{2}\right|}{N}$$

To measure the cumulative error between the predicted (x), and the experimental datasets (y) and the Normalized Root Squared Error (NRSE)

2 $$NRS{E}_{n}= \frac{\sqrt{{(x}_{n}-{y}_{n}{)}^{2}}}{MAX. RSE}$$

between each data point. The multiplexed phosphoproteomics measurements by Melas et al.^12^ and Neidlin et al.^13^ were used for calibration and independent validation purposes, respectively. Finally, to evaluate how our model could integrate/assimilate metabolism changes, we assessed the ability to predict significantly different SSs under different initial conditions through a t-test (α = 0.05).

### Literature-based (LIT) network

First, we collected OA- and chondrocyte-related protein–protein interactions based on the careful examination of a corpus of 63 expert journal articles (information summarized in Additional Material A1 Table 3). As a result, a first chondrocyte-specific model was developed based on the literature. We gathered information from different databases by using the keywords “osteoarthritis” AND “chondrocyte” AND “the molecule/node studied”. Only peer-reviewed articles from indexed journals were retained. We considered the action of important proinflammatory cytokines (interleukin 1 beta (IL-1β), tumor necrosis factor-alpha (TNF-α), interleukin 6 (IL-6), leukemia inhibitory factor (LIF), interleukin 17 (IL-17) and interleukin 18 (IL-18)), chemokines (interleukin 8 (IL-8)) and interferon gamma (IFN-γ). Proanabolic growth factors were also included: transforming growth factor beta (TGF-β), fibroblast growth factor (FGF_2_) and bone morphogenic protein-2. (BMP_2_). They were studied at the cellular level with respect to the self-expression of matrix degrading enzymes (matrix metalloproteinases (MMPs) and a desintegrin-like and metalloproteinase with thrombospondin (ADAMTs)), structural proteins (collagen type II (COL2A) and aggrecan (ACAN)), pain-related factors, such as vascular endothelial growth factor (VEGF), and tissue inhibitor of metalloproteinases (TIMP). Intermediate metabolites were not included to facilitate model comparisons with cell/tissue culture experimental data. Based on Additional Material A1 Table 3 information, the LIT model was represented as a directed static graph, where a set of nodes, i.e., molecules, were related to each other through activating and inhibiting edges. To understand their directionality, the role of each node in the context of chondrocyte metabolism is summarized in Additional Material A1 Table 1 in the additional material.

### Network solving: the stable steady states

The activation of each node depends on the initial conditions imposed on the system of ordinary differential equations. To calculate the final expression rate of each node, the static graph was mathematically translated into a semiquantitative model through a set of ordinary differential equations. For a given topology, the network state, *S*, at time *t* is the set of all node activation values at time *t*:

3 $${\mathrm{S}}\left(t\right)=\left\{{x}_{1}\left(t\right),{x}_{2}\left(t\right),\dots, {x}_{N}\left(t\right)\right\},$$

which can be considered a point in the (N-dimensional) state space of the system. If the state changes with time, the model is dynamic, and the time-varying components of the state can be defined as x_n_(t) with n = $\left\{1,\dots ,N\right\}$. Then, the future state given an initial past state can be expressed as:

4 $${\mathrm{S}}\left({t}_{2}\right){\mathrm{f}}=\left(\left\{S{\left(t\right\}}_{1}\right);{\mathrm{p}}\right), \quad {t}_{2}>{t}_{1}$$

where *f* is a function that encodes the topology and the kinetics of the interactions among the components of the system and depends on the current state of the network as well as on the time-independent parameters, *p*. If the normalized concentration of each node, x_n_, is the unique continuous time-dependent variable of the system, ordinary differential equations (ODEs) can be used to define the rates of change of the system components and, therefore, the future states of the network:

5 $$\frac{d{x}_{n}}{dt}\left(t\right)={\mathrm{f}}\left(\left\{{x}_{n}\left(t\right)\right\},p\right)$$

Generally, *f* in biochemical reactions follows Michaelis–Menten law^43^. However, it requires the complete signaling route and its reaction rate constants. Since this information is hard to find, *f* was defined following the method developed by Mendoza et al.^25^, which allows the modeling of key molecular player interactions in the midst of complex regulatory processes without taking into account the governing biochemical reactions. For node *n*, let *x*_*n*_ ∈ [0, 1] denote its (normalized) activation level; let {${x}_{np}^{a}$}, p = 1, …, P_n_, and ${\{x}_{nk}^{i}$}, k = 1,…,K_n_, be the (levels of the) set of activators and inhibitors, respectively. Then, the total input ω_n_ to the node is a combination of activation and inhibitory inputs:

6 $$\begin{aligned} {\upomega }_{\mathrm{n}} &= \left\{\begin{array}{l}\left(\frac{1+\sum {{\upalpha }}_{np}}{\sum {{\upalpha }}_{np}}\right)\left(\frac{\sum {\alpha }_{np}{x}_{np}^{a}}{1+\sum {\alpha }_{np}{x}_{np}^{a}}\right)\left(1-\left(\frac{1+\sum {\beta }_{nk}}{\sum {\beta }_{nk}}\right)\left(\frac{\sum {\beta }_{nk}{x}_{nk}^{i}}{1+\sum {\beta }_{nk}{x}_{nk}^{i}}\right)\right) {\mathfrak{J}}\\ \left(\frac{1+\sum {\alpha }_{np}}{\sum {\alpha }_{np}}\right)\left(\frac{\sum {\alpha }_{np}{x}_{np}^{a}}{1+\sum {\alpha }_{np}{x}_{np}^{a}}\right) {\mathfrak{JJ}} \\ \left(1-\left(\frac{1+\sum {\beta }_{nk}}{\sum {\beta }_{nk}}\right)\left(\frac{\sum {\beta }_{nk}{x}_{nk}^{i}}{1+\sum {\beta }_{nk}{x}_{nk}^{i}}\right)\right) {\mathfrak{JJJ}}\end{array}\right.\\ &\qquad 0 \le x_{n} \le 1\\&\qquad 0 \le {\upomega }_{n} \le 1 \\ &\qquad h_{n} ,{\upalpha }_{np} ,{\upbeta }_{nk} ,{\upgamma }_{n} > 0 \\&\qquad x_{np}^{a} {\text{ is the set of activators of }}x_{n} \\ &\qquad x_{nk}^{i} {\text{ is the set of inhibitors of }}x_{n} \\ &\qquad {\mathfrak{J}} \;{\text{is}}\;{\text{used}}\;{\text{if}}\;x_{n} \;{\text{has}}\;{\text{activators}}\;{\text{and}}\;{\text{inhibitors}}\\ &\qquad {\mathfrak{JJ}} \;{\text{is}}\;{\text{used}}\;{\text{if}}\;x_{n} \;{\text{has}}\;{\text{only}}\;{\text{activators}} \\ &\qquad {\mathfrak{JJJ}} \;{\text{is}}\;{\text{used}}\;{\text{if}}\;x_{n} \;{\text{has}}\;{\text{only}}\;{\text{inhibitors}} \end{aligned}$$

where ${{\upalpha }}_{np}>0$ and ${\upbeta }_{nk}>0$ are the weights of the corresponding activators and inhibitors, respectively. Note that ω_n_ ∈ [0, 1] since *x*_*n*_ ∈ [0, 1] and the activator and inhibitor weights are positive. The regulatory network can then be described by

7 $$\frac{d{x}_{n}}{dt}=\frac{-{e}^{0.5{h}_{n}}+{e}^{-{h}_{n}\left({\upomega }_{n}-0.5\right)}}{\left(1-{e}^{0.5{h}_{n}}\right)\left(1+{e}^{-h\left({\upomega }_{n}-0.5\right)}\right)}-{\upgamma }_{n}{x}_{n}$$

where the first term of the right-hand side of Eq. (5) represents an activation function (i.e., a sigmoid function, with gain h_n_, of the total input ω_n_) and the second term is the decay, which is proportional to the activation level of the node by a factor γ_n_ > 0.

As proposed by Mendoza et al.^25^, we set the activator and inhibitor weights to 1, i.e., ${{\upalpha }}_{np}={\upbeta }_{nk}=1$ for all n, p, k, and chose h_n_ = 10 and γ_n_ = 1 for all n. These values were confirmed by Feher et al.^44^ to be a suitable choice, since they ensure a smooth sigmoidal response that better reproduces the biological response of a cell to an external stimulus.

The system was solved through the Runge–Kutta (order 4) method in MATLAB 2018b (The MathWorks, Inc, Massachusetts, USA) with the ode45 solver to find a global attractor or an steady state (SS) (see Fig. 8B). SS shall correspond to patterns of cell expression that can stand for systematic descriptions of chondrocyte regulation by microenvironmental chemokines. This theoretical information can be directly compared to experimental measurements from chondrocyte cultures and cartilage tissue explants.

### Baseline calculations

A Steady State (SS) baseline was calculated for each network by a random initial condition based on Mersenne Twistter 0 seeds. To assess the effects of the imposed perturbations, the SS baseline was compared to the perturbed SS through t-test calculations. Furthermore, the subtraction of the basal SS to the perturbed SS for quantitative network calibrations and validations made our theoretical node data range between − 1 and 1, which was extremely convenient for direct comparisons with the experimental data.

### Enriched (ENR) network

For network enrichment, we used STRING (version 11.0)^21^ to complement the network with new interactions that could not be found in the literature review. First, we looked for the UniProt identification number of the human type of each protein in the literature-based (LIT) interactome. Then, in STRING^21^, we used the following list downloaded directly from UniProt as input to the multiple proteins section: P02458, P10145, P05231, Q14116, P16112, O75173, P01137, P50281, P45452, P03956, P01375, Q16552, P22301, P35225, P05112, P12643, P05019, P09038, P99999, Q14790, P01033, P15692, P01584, P08254. We selected Homo sapiens as the organism. In the setting section, we selected text mining, experiments and databases with a minimum required interaction score with a medium confidence of 0.400. As the latter ensured inclusiveness, the automated data retrieval was followed by a second step of manual curation, to eliminate irrelevant or unrelated data. The stable SS of the ENR model was solved by using the system of Eq. (4), which was also used for the LIT network.

### Optimized (OPT) network

Experimental information from Melas et al.^12^ was used to optimize the topology of the ENR model. Data consisted of cytokine release measurements with a Luminex assay (normalized between − 1 and 1) after different biochemical OA-related perturbations of monolayer chondrocyte cultures. We established the absolute mean difference (AMD) between the log-normalized data sets as fitness functions:

6 $$AMD=\left|\frac{\sum_{n}^{N}({\mathrm{ln}}(x_{n}+2))-{\mathrm{ln}}(y_{n}+2))}{N}\right|$$

where the data shift by 2 ensures that the range of the logarithm starts at 0 (avoiding negative and asymptotic values) between the predicted (x) and the experimental (y) data sets. We chose to log-normalize the data to penalize higher percentual differences.

Function (6) was minimized through a genetic algorithm (GA) configured with default parameters in the MATLAB-GA toolbox^45^ except for the vectorization option, which was enabled. GA is a method for solving optimization problems based on a natural selection process that mimics biological evolution. At each step or generation, the algorithm selects individuals based on their efficiency (defined by an objective function that will define the best adapted individuals within a population) and a random component that replicates the randomness in genetic mutations in species. GA will use the selected individuals as parents to produce the children for the next generation. Over successive generations, the population “evolves” toward an optimal solution. The best point in the population in terms of the objective function approaches an optimal solution.

The solution found by the genetic algorithm (GA) led to a new network topology, i.e., the OPT network. Since GA’s random component, we repeated the optimization step 100 times, so we have generated 100 optimized networks. The selected final optimized network was chosen based on a careful examination of both the qualitative and quantitative results (see supplementary material) reviewed in the “Discussion” section. In addition to Function (6), we evaluated seven fitness functions (see Additional Material 2) with and without additional qualitative-based constraints. This constraint aims to penalize those with a catabolic baseline. Overall, a total of 1800 networks were explored.

### Qualitative assessment

We would like to assess the capacity of our model to represent current biological knowledge, not common assessed by computational models that are mainly focused on quantitative validations. Then, two evaluations has been done:

#### Simulation of expected anabolic/catabolic responses

The OPT, ENR and LIT models were qualitatively evaluated against their capacity to converge toward an anabolic SS without any imposed initial conditions (this could be understood as the capacity to represent a healthy chondrocyte by our network model). Then, we tested their ability to replicate literature-established behaviors of chondrocytes under different pro-catabolic/anabolic microenvironments: we looked whether our model could reproduce reported chondrocyte behaviors in articular cartilage in the presence of single pro- or anti-inflammatory perturbations (summarized in Additional Material A1 Table 2 of the additional material).

#### Treatment simulation

Many hemoderivative treatments for OA have high potential to modulate OA progression. We have selected Frizziero et al.^14^ study due to its high number of patients involved (n = 376), i.e., the largest number of participants implicated for this type of OA treatment as far as we are aware. Authors described that the injection of autologous conditioned serum (ACS), enriched with TGF-β, IGF_1_, IL-4, IL-10 and IL-1β antagonist, can significantly reduce pain in OA compared to placebo patients (*p* < 0.001).

Treatment simulations were combined with the definition of three different possible scenarios for concurrent synovitis. First, the OPT network was properly preconditioned to simulate an OA joint to achieve OA-like SS: proinflammatory cytokine stimulation (IL-1β, TNF-α, IL-6, IL-8, IL-17 and IL-18). Then, the three different scenarios were:

- Scenario 1: Catabolic nodes (but IL-1β) were free to be regulated by the network calculations. This scenario simulated the full potential of ACS molecules to regulate the inflammation that comes from the synovium.
- Scenario 2: In a persistent OA environment because of sustained synovitis, we clamped the pro-catabolic nodes at 1, in addition to the fixed activation of the ACS nodes and inhibition of IL-1β. The subjacent assumption is that the synovium continues to send catabolic signals to chondrocytes.
- Scenario 3: Proanabolic molecules from the ACS were clamped at 1, and the catabolic nodes (but IL-1β) were clamped at 50% of their potential. This scenario assumed that the ACS molecules would mitigate the inflammation of the synovium and that their boundary concentrations would be higher than those of the pro-catabolic molecules.

t-tests (α = 0.05) were applied to check whether there was a significant difference between the OA-like SS state and the resulting state after the virtual administration of the ACS.

## Supplementary Information

Supplementary Information 1.

Supplementary Information 2.

Supplementary Information 3.

## Abbreviations

OA: Osteoarthritis
SS: Steady states
GA: Genetic algorithm
NMAD: Normalized mean absolute deviation
NRSE: Normalized root squared error
LIT: Literature-based network
AMD: Absolute mean deviation
ENR: Enriched network
OPT: Optimized network
ACS: Autologous conditioned serum
IL-1β: Interleukin 1 β
TNF-α: Tumor necrosis factor-alpha
IL-6: Interleukin 6
LIF: Leukemia inhibitory factor
IL-17: Interleukin 17
IL-18: Interleukin 18
IL-8: Interleukin 8
IFN-γ: Interferon gamma
TGF-β: Transforming growth factor beta
FGF_2_: Fibroblast growth factor-2
BMP_2_: Bone morphogenic protein-2
MMPs: Matrix metalloproteinases
ADAMTs: A desintegrin-like and metalloproteinase with thrombospondin
COL2A: Collagen type II
ACAN: Aggrecan
VEGF: Vascular endothelial growth factor
PEG2: Prostaglandin E2
