# Supplementary material for: Regulatory network-based model to simulate the biochemical regulation of chondrocytes in healthy and osteoarthritic environments
Source: Sci Rep. 2022 Mar 9;12:3856. doi: 10.1038/s41598-022-07776-2 (PMC8907219; doi:10.1038/s41598-022-07776-2)
Supplement: Supplementary file 1 — Supplementary Information 1. [file 41598_2022_7776_MOESM1_ESM.pdf]

Additional Material 1. Knowledge-driven tables

In A1 Table 1 are summarized the roles of each molecule on chondrocyte metabolism. The second A1 Table 2 are referenced the responses used to assess the networks qualitatively. Finally, in A1 Table 3 can be seen the references of each interaction for the literature-based network (LIT) and the enriched network (ENR).

A1 Table 1. Role of the molecules on chondrocyte metabolism.

Molecule	*Action on chondrocyte metabolism*
ACAN	It is a proteoglycan with chondroitin-sulphate and keratan-sulphate chains that gives articular cartilage with its ability to handle compressive loads [1]
ADAMTs	The main role of this enzyme is to cleave ACAN [1]
BMP_2	It increases chondrocyte anabolism, but also catabolism to let pass new structural proteins. Then it is one of the last attempts of chondrocyte to repair AC. [2]
CASP8	When its levels are increased, it means that apoptosis has begun in chondrocytes. [3]
COL2A	It is the main structural protein that conforms articular cartilage. [4]
CYCS	When its levels are increased, it means that apoptosis has begun in chondrocytes. [3]
FGF_2	It is a grow factor with opposite functions. Regarding the nature of receptor that will bind, it will have catabolic or anabolic effects on chondrocytes. [5]
IFN-γ	IFN-γ stimulates the production of IL-6, IL-1ra, NO and PGE_2 and inhibit PG synthesis[6]
IGF_1	IGF_1 increases when osteophyte is formed. No evident results that IGF_1 helps in the prevention of cartilage degradation. So, IGF_1 is higher in late OA, because osteophytes are formed at the last steps of this disease. [7]
IL-10	It inhibits IL-1β and TNF-α expression.[8]
IL-13	It inhibits the inflammatory processes, reduces the secretion of inflammatory cytokines and metalloproteinases, while stimulating the synthesis of IL-1Ra, a chondroprotective receptor.[9]
IL-17	It increases the expression of IL-1β, TNF-α,IL-6 and NOs.[10,11]
IL-18	NO and IL-6 increase when IL-18 is present. IL-18 inhibits cell proliferation induced by TGF-β. It suppresses proteoglycan synthesis, inhibition of chondrocyte migration and induces chondrocyte apoptosis.[12]
IL-1β	IL-1β is associated with cartilage destruction. It suppresses type II collagen and ACAN expression, stimulates the release of MMP-, MMP3 and MMP13. It induces the production of IL-6 and IL-8. [99]
IL-4	IL-4 has a chondroprotective effect by inhibiting the secretion of MMPs, besides lowering the production of proteoglycans.[9]
IL-6	It induces an amplification of the IL-1β effects on the MMPs synthesis. It Inhibits proteoglycan production [15] and induces TIMP because it is believed that this cytokine is involved in the feedback mechanism that limits enzyme damage. [16]
IL-8	It induces NOs, MMP1 and IL-6, and proteoglycan depletion[35]
LIF	It stimulates cartilage proteoglycan, MMPs and NOs[15]
MMPs	They degrade proteoglycans and collagen of the extracellular matrix. They are inhibited by TIMP.[15]
NO	Synthetized by nitric oxide synthase (Nos), it inhibits the synthesis of collagen and induces apoptosis.[17]
PGE_2	It is an inflammatory marker pain related. It is synthetized by cyclooxygenase and prostaglandin E synthase. [18]
TGF-β	It is possible that TGF-β isoform expression increases in the early stages OA in an attempt to counteract the catabolic effects of inflammatory cytokines or as an adaptive response to the progressive loss of TGF-β receptor expression but then decreases. [19,20]
TIMP	It maintains the low turnover of chondrocytes and homeostasis of articular cartilage [21]
TNF-α	It drives the pro-inflammatory cascade by increasing the expression of other cytokines. [15]
VEGF	An hypertrophy marker, its expression correlates with disease severity. [22]

A1 Table 2. Expected outcomes from chondrocytes under single stimulations used to assess qualitatively our three networks. A response marked with a ⇑ means that the stimuli might increase its expression. Otherwise, if it is marked with ⇓, its expression might decrease.

Stimuli	***Expected responses***								
TNF-α	⇑CYCS[23]	⇑IL-8[24]	⇑NO[25]	⇓COL2A[15,26]	⇓ACAN[15]	⇑MMP1[15]	⇑MMP3[15]	⇑MMP1[15]	⇑IL-6[15]
IL-1β	⇑IL-6[24]	⇑PGE_2[24]	⇑NO[15]	⇓COL2A[15]	⇓ACAN[15]	⇑MMP1[15]	⇑MMP3[15]	⇑MMP1[15]	⇑IL-8[24]
IL-6	⇓TIMP[24]	⇑MMP1[24]	⇓COL2A[15]	⇓ACAN[15]					
IL-8	⇑TNF-α [24]	⇑IL-1β[24]	⇑IL-6[24]	⇑NO[15]	⇑MMP1[15]	⇓ACAN[15]			
IL-17	⇑IL-1β[24]	⇑TNF-α [24]	⇑IL-6[24]	⇑MMPs[24]	⇑NO[15]	⇓ACAN[15]			
LIF	⇑TNF-α [24]	⇑IL-1β[24]	⇓ACAN[15]	⇑MMPs[15]	⇑NO[24]				
NO	⇑IL-18[25]	⇑MMPs[25,27]	⇑CYCS[25,27]						
BMP2	⇑COL2A[26]	⇑ACAN[26]	⇑MMP13[28]						
INF-γ	⇓COL2A[26]	⇓ACAN[29]							
PGE_2	⇑VEGF[30]								
IGF_1	⇑COL2A[23]	⇑ACAN[23]							
TGF-β	⇑COL2A[23]	⇑ACAN[23]							
FGF_2	⇑COL2A[23]	⇑ACAN[23]							
IL-4	⇓IL-1β[15]	⇓TNF-α[15]	⇓MMPs[15]	⇑TIMP[15]					
IL-10	⇓IL-1β[15]	⇓TNF-α [15]	⇓MMPs[15]	⇑TIMP[15]					
IL-13	⇓IL-1β[15]	⇓TNF-α [15]	⇓MMPs[15]	⇑TIMP[15]					

*A 1 Table 3. Summarized interactions of the OA literature-based network (LIT) and the enriched network (ENR). Each node of the network is listed in the column Nodes. At its right, you can find listed the inputs of each node separated by activating and inhibiting links. Edges marked with * are found by STRING[31] but are not chondrocyte restricted (mainly extracted from curated databases), links market with [⊗] are added in the enriched model, but they are OA related.*

Nodes	*Activators*	*Inhibitors*
ACAN	IL-4[32],TGF-β[19,20],BMP2[33,34],IL-10[35], IGF_1[36⊗]	IL-1β [37], TNF-α [38], NO[38], IL-6[39], MMP14[21], PGE_2 [40]
ADAMTs	IL-1β [41],TNF-α[42],NO[38],IL6[43]	TGF-β[44]
BMP_2	IL-1β [2⊗],TNF-α[2⊗]	0
CASP8	TNF-α[45],NO[17]	IL-10[9]
COL2A	IL-10[35], BMP2[34], IGF_1[46], TGF-β[20],IL4[47]	IL-6[48], IL-1β [49],TNF-α[50],NO[17]
CYCS	NO[17]	IL-10[8]
FGF_2	0	0
IFN-γ	0	IL4[51]
IGF_1	IL-4[47]	IL1β [52],IL6[53]
IL-10	IL-4[54]*	0
IL-13	IL-4[54]*	TNF-α[55]*
IL-17	0	IL-4[56,57],IL-10[58],IL-13[59]
IL-18	IL-1β [60],NO[17]	IL-10[61],IL-13[62],IL-4[62]
IL-1β	TNF-α[49],LIF [16],IL-17 [63,64]	IL-10[28], IL-4[47],TGF-β[19], BMP_2[33],IL-13[65]
IL-4	TGF-β[66]*	0
IL-6	IL-1β[16,67], TNF-α[16,67], LIF[16], IL-17[11], IL-18 [12,60], IL-6[16], IL-8[16]	IL-4[47],IL-10[68],IL-13[69]
IL-8	IL-1β [16,49],TNF-α[16],LIF[16],IL-17[11],IL-6[16]	IL-10[70],IL-4[71],IL-13[69],IL-6[16]
LIF	IL-1β [16],TNF-α[16],IL-6[16]	0
MMP1	IL-1β [49,72],TNF-α[21],IL-17[73],NO[74], IL-18[60]	TIMP[11,57],IL-13[76]*,TGF-β[77],
MMP13	IL-1β[49,78], TNF-α [21], IL-17[11], NO[74], IL-8[79], IFN-γ[80], MMP14[21], BMP_2[28], IL-18[60]	TIMP[11,57] ,IL-13[81]*
MMP14	IL-1β,NO, IL-18[60]	IL-10[82]
MMP3	IL-1β [49,72],TNF-α[83],IL-17[11],NO[74], IL-18[60]	TIMP[11,57] ,IGF_1[84],TGF-β[85], IL-4[86]
NO	IL-1β [49],TNF-α[87],IL-17[88],IL-18[60], IFN-γ[89]	IL-4[90]
PGE_2	IL-1β [49],TNF-α[18],IL-18[60]	IL-4[47], IL-13[82]
TGF-β	0	TNF-α[91*92⊗], IL-1β [92⊗]
TIMP	IL-6[21],IL-10[93],IGF_1[94],TGF-β[95]	TNF-α[96],NO[17]
TNF-α	IL-1β [97],IL-17[11],IL-6[16]	IL-10[98]*,IL-4[69]*,IL-13[65]*
VEGF	PEG2[22],MMP3[21],ADAMTs[21],MMP13[21]	IL-13[99]*

A 1 Table 4. Summarized interactions of the optimized network. Each node of the network is listed in the column Nodes. At its right, you can find listed the inputs of each node separated by activating and inhibiting links.

Nodes	*Activators*	*Inhibitors*
ACAN	BMP_2, IGF_1, IL-10, IL-4, TGF-β	PEG_2, TNF-α
ADAMTs	IL-6,NO	0
BMP_2	0	0
CASP8	NO	IL-10
COL2A	IGF_1, IL-10	IL-1β, NO, TNFα
CYCS	NO	IL-10
IFN-γ	0	0
IGF_1	0	IL-1β, IL-6
IL-10	IL-4	0
IL-13	IL-4	0
IL-17	0	0
IL-18	IL-1β, NO	IL-13
IL-1β	LIF, TNF-α	BMP_2, IL-13, IL-4, TGF-β
IL-4	0	0
IL-6	IL-8	IL-10, IL-13
IL-8	IL-1β, LIF, TNF-α	IL-10, IL-13, IL-6
LIF	0	0
MMP1	IL-1β	TIMP
MMP13	BMP_2, IL-17, IL-18, IL-1β, IL-8, TNF-α	IL-13
MMP14	IL-18, NO	IL-10
MMP3	NO, TNF-α	TGF-β
NO	IFN-γ, IL-1β,TNF-α	IL-4
PGE_2	IL-18, IL-1β	0
TGF-β	IL-1β, TNF-α	0
TIMP	0	TNF-α
TNF-α	IL-6, TGF-β	TNF-α
VEGF	IL-17, IL-1β, IL-6	IL-10, IL-13

References

1. Roughley PJ, Mort JS. The role of aggrecan in normal and osteoarthritic cartilage. *Journal of experimental orthopaedics*. 2014;1(1):8. doi:10.1186/s40634-014-0008-7

2. Fukui N, Zhu Y, Maloney WJ, Clohisy J, Sandell LJ. Stimulation of BMP-2 expression by pro-inflammatory cytokines IL-1 and TNF-α in normal and osteoarthritic chondrocytes. In: *Journal of Bone and Joint Surgery - Series A*. Vol 85. Journal of Bone and Joint Surgery Inc.; 2003:59-66. doi:10.2106/00004623-200300003-00011

3. McCulloch RS, Ashwell MS, Maltecca C, O'Nan AT, Mente PL. Progression of Gene Expression Changes following a Mechanical Injury to Articular Cartilage as a Model of Early Stage Osteoarthritis. *Arthritis*. 2014;2014:371426. doi:10.1155/2014/371426

4. Sulzbacher I. Osteoarthritis: Histology and pathogenesis. *Wiener Medizinische Wochenschrift*. 2013;163(9-10):212-219. doi:10.1007/s10354-012-0168-y

5. Vincent TL. Fibroblast growth factor 2: good or bad guy in the joint? *Arthritis research & therapy*. 2011;13(5):127. doi:10.1186/ar3447

6. Henrotin YE, Zheng SX, Labasse AH, Deby GP, Crielaard JMR, Reginster JYL. Modulation of human chondrocyte metabolism by recombinant human interferon. *Osteoarthritis and Cartilage*. 2000;8(6):474-482. doi:10.1053/joca.1999.0323

7. Michael JWP, Schlüter-Brust KU, Eysel P. Epidemiologie, ätiologie, diagnostik und therapie der gonarthrose. *Deutsches Arzteblatt*. 2010;107(9):152-162. doi:10.3238/arztebl.2010.0152

8. Iannone F, De Bari C, Dell'Accio F, et al. Interleukin-10 and interleukin-10 receptor in human osteoarthritic and healthy chondrocytes. *Clinical and experimental rheumatology*. 2001;19(2):139-145. http://www.ncbi.nlm.nih.gov/pubmed/11332442. Accessed June 11, 2019.

9. Wojdasiewicz P, Poniatowski ŁA, Szukiewicz D. The role of inflammatory and anti-inflammatory cytokines in the pathogenesis of osteoarthritis. *Mediators of inflammation*. 2014;2014:561459. doi:10.1155/2014/561459

10. Martel-Pelletier J, Mineau F, Jovanovic D, Di Battista JA, Pelletier J-P. Mitogen-activated protein kinase and nuclear factor ?B together regulate interleukin-17-induced nitric oxide production in human osteoarthritic chondrocytes: Possible role of transactivating factor mitogen-activated protein kinase-activated protein kinase (MAPKAPK). *Arthritis & Rheumatism*. 1999;42(11):2399-2409. doi:10.1002/1529-0131(199911)42:11<2399::AID-ANR19>3.0.CO;2-Y

11. Onishi RM, Gaffen SL. Interleukin-17 and its target genes: Mechanisms of interleukin-17 function in disease. *Immunology*. 2010;129(3):311-321. doi:10.1111/j.1365-2567.2009.03240.x

12. Olee T, Hashimoto S, Quach J, Lotz M. IL-18 is produced by articular chondrocytes and induces proinflammatory and catabolic responses. *Journal of immunology (Baltimore, Md : 1950)*. 1999;162(2):1096-1100. http://www.ncbi.nlm.nih.gov/pubmed/9916738. Accessed June 11, 2019.

13. Chadjichristos C, Ghayor C. Sp1 and Sp3 Transcription Factors Mediate Interleukin-1 beta Down-regulation of Human Type II Collagen Gene Expression in Articular Chondrocytes. *Journal of Biological Chemistry*. 2003;278(41):39762-39772. doi:10.1074/jbc.M303541200

14. Lotz M, Terkeltaub R, Villiger PM. Cartilage and joint inflammation. Regulation of IL-8 expression by human articular chondrocytes. *Journal of immunology (Baltimore, Md :*

1950). 1992;148(2):466-473. http://www.ncbi.nlm.nih.gov/pubmed/1729366. Accessed June 11, 2019.

15. Kapoor M, Martel-Pelletier J, Lajeunesse D, Pelletier JP, Fahmi H. Role of proinflammatory cytokines in the pathophysiology of osteoarthritis. *Nature Reviews Rheumatology*. 2011;7(1):33-42. doi:10.1038/nrrheum.2010.196

16. Zheng S, Geenen VG, Reginster JL. Effects of exogenous IL-1b, TNF-a, IL-6, IL-8 and LIF cytokine production by human articular chondrocytes. *Osteoarthritis and Cartilage*. 1996;4:163-173.

17. Abramson SB. Osteoarthritis and nitric oxide. *Osteoarthritis and Cartilage*. 2008;16(SUPPL. 2):S15-S120. doi:10.1016/S1063-4584(08)60008-4

18. Masuko K, Yudoh K, Nakamura H. *Expression of Prostaglandin E2 Receptors in Chondrocytes: A Potential Therapeutic Target in the Treatment of Osteoarthritis?* Vol 10.; 2009.

19. Blaney Davidson EN, van der Kraan PM, van den Berg WB. TGF-β and osteoarthritis. *Osteoarthritis and Cartilage*. 2007;15(6):597-604. doi:10.1016/j.joca.2007.02.005

20. Zhu Y, Tao H, Jin C, et al. Transforming growth factor-β1 induces type II collagen and aggrecan expression via activation of extracellular signal-regulated kinase 1/2 and Smad2/3 signaling pathways. *Molecular medicine reports*. 2015;12(4):5573-5579. doi:10.3892/mmr.2015.4068

21. Rose BJ, Kooyman DL. A Tale of Two Joints: The Role of Matrix Metalloproteases in Cartilage Biology. *Disease Markers*. 2016;2016. doi:10.1155/2016/4895050

22. Timur U, Caron M, Bastiaansen-Jenniskens Y, Welting T, van Osch G, Emans P. PGE2 and PGF2α are secreted by the osteoarthritic infrapatellar fat pad and their release can be modulated by celecoxib. *Osteoarthritis and Cartilage*. 2016;24:S339. doi:10.1016/j.joca.2016.01.608

23. Goldring MB, Otero M, Tsuchimochi K, Ijiri K, Li Y. Defining the roles of inflammatory and anabolic cytokines in cartilage metabolism. *Annals of the rheumatic diseases*. 2008;67 Suppl 3(Suppl 3):iii75-82. doi:10.1136/ard.2008.098764

24. Fernandes JC, Martel-Pelletier J, Pelletier J-P. The role of cytokines in osteoarthritis pathophysiology. *Biorheology*. 2002;39(1-2)(237):46.

25. Abramson SB. Osteoarthritis and nitric oxide. *Osteoarthritis and cartilage*. 2008;16 Suppl 2:S15-20. doi:10.1016/S1063-4584(08)60008-4

26. Goldring MB, Otero M, Plumb DA, et al. Roles of inflammatory and anabolic cytokines in cartilage metabolism: signals and multiple effectors converge upon MMP-13 regulation in osteoarthritis. *European cells & materials*. 2011;21:202-220. doi:10.22203/ecm.v021a16

27. Aigner T, McKenna L. Molecular pathology and pathobiology of osteoarthritic cartilage. *Cellular and Molecular Life Sciences (CMLS)*. 2002;59(1):5-18. doi:10.1007/s00018-002-8400-3

28. Nakashima A, Tamura M. Regulation of matrix metalloproteinase-13 and tissue inhibitor of matrix metalloproteinase-1 gene expression by WNT3A and bone morphogenetic protein-2 in osteoblastic differentiation. *Frontiers in Bioscience*. 2006;11(2 P.1591-2006):1667-1678. doi:10.2741/1912

29. Verbruggen G, Malfait AM, Veys EM, Gyselbrecth L, Lambert J, Almqvist KF. Influence of interferon-gamma on isolated chondrocytes from human articular cartilage.

Dose dependent inhibition of cell proliferation and proteoglycan synthesis - PubMed. *J Rheumatol*. 1993:1020-1026. https://pubmed.ncbi.nlm.nih.gov/8350308/. Accessed February 16, 2021.

30. Huang SP, Wu MS, Shun CT, et al. Cyclooxygenase-2 increases hypoxia-inducible factor-1 and vascular endothelial growth factor to promote angiogenesis in gastric carcinoma. *Journal of Biomedical Science*. 2005;12(1):229-241. doi:10.1007/s11373-004-8177-5

31. Jensen LJ, Kuhn M, Stark M, et al. STRING 8 - A global view on proteins and their functional interactions in 630 organisms. *Nucleic Acids Research*. 2009;37(SUPPL. 1). doi:10.1093/nar/gkn760

32. Shioji S, Imai S, Ando K, Kumagai K, Matsusue Y, Nurminsky DI. Extracellular and intracellular mechanisms of mechanotransduction in three- Dimensionally embedded rat chondrocytes. *PLoS ONE*. 2014;9(12):1-13. doi:10.1371/journal.pone.0114327

33. Gamer L, Cox K, Lin Q, Han L, Rosen V. Role of BMP2 in the maturation and maintenance of the knee joint. *Osteoarthritis and Cartilage*. 2015;23:A56-A57. doi:10.1016/j.joca.2015.02.119

34. Kohno Y, Mizuno M, Ozeki N, et al. Comparison of mesenchymal stem cells obtained by suspended culture of synovium from patients with rheumatoid arthritis and osteoarthritis. *BMC Musculoskeletal Disorders*. 2018;19(1). doi:10.1186/s12891-018-1998-6

35. Behrendt P, Preusse-Prange A, Klüter T, et al. IL-10 reduces apoptosis and extracellular matrix degradation after injurious compression of mature articular cartilage. *Osteoarthritis and Cartilage*. 2016;24(11):1981-1988. doi:10.1016/j.joca.2016.06.016

36. Darling EM, Athanasiou KA. Growth factor impact on articular cartilage subpopulations. *Cell and Tissue Research*. 2005;322(3):463-473. doi:10.1007/s00441-005-0020-4

37. Demoor-Fossard M, Redini F, Boittin M, Pujol JP. Expression of decorin and biglycan by rabbit articular chondrocytes. Effects of cytokines and phenotypic modulation. *Biochimica et biophysica acta*. 1998;1398(2):179-191. doi:10.1016/s0167-4781(98)00044-x

38. Voigt H, Lemke AK, Mentlein R, Schünke M, Kurz B. Tumor necrosis factor alpha-dependent aggrecan cleavage and release of glycosaminoglycans in the meniscus is mediated by nitrous oxide-independent aggrecanase activity in vitro. *Arthritis Research & Therapy*. 2009;11(5):R141. doi:10.1186/ar2813

39. Legendre F, Bogdanowicz P, Boumediene K, Pujol J-P. Role of interleukin 6 (IL-6)/IL-6R-induced signal tranducers and activators of transcription and mitogen-activated protein kinase/extracellular. *The Journal of rheumatology*. 2005;32(7):1307-1316. http://www.ncbi.nlm.nih.gov/pubmed/15996070. Accessed November 18, 2019.

40. Li X, Ellman M, Muddasani P, et al. Prostaglandin E2 and its cognate EP receptors control human adult articular cartilage homeostasis and are linked to the pathophysiology of osteoarthritis. *Arthritis and rheumatism*. 2009;60(2):513-523. doi:10.1002/art.24258

41. Stradner MH, Hermann J, Angerer H, et al. Spingosine-1-phosphate stimulates proliferation and counteracts interleukin-1 induced nitric oxide formation in articular chondrocytes. *Osteoarthritis and Cartilage*. 2008;16(3):305-311. doi:10.1016/j.joca.2007.06.018

42. Xue J, Wang J, Liu Q, Luo A. Tumor necrosis factor-α induces ADAMTS-4 expression in human osteoarthritis chondrocytes. *Molecular Medicine Reports*. 2013;8(6):1755-

1760. doi:10.3892/mmr.2013.1729

43. MIMATA Y, KAMATAKI A, OIKAWA S, et al. Interleukin-6 upregulates expression of ADAMTS-4 in fibroblast-like synoviocytes from patients with rheumatoid arthritis. *International Journal of Rheumatic Diseases*. 2012;15(1):36-44. doi:10.1111/j.1756-185X.2011.01656.x

44. Thielen NGM, van der Kraan PM, van Caam APM. TGFβ/BMP Signaling Pathway in Cartilage Homeostasis. *Cells*. 2019;8(9):969. doi:10.3390/cells8090969

45. López-Armada MJ, Caramés B, Lires-Deán M, et al. Cytokines, tumor necrosis factor-alpha and interleukin-1beta, differentially regulate apoptosis in osteoarthritis cultured human chondrocytes. *Osteoarthritis and cartilage*. 2006;14(7):660-669. doi:10.1016/j.joca.2006.01.005

46. Zhang M, Zhou Q, Liang QQ, et al. IGF-1 regulation of type II collagen and MMP-13 expression in rat endplate chondrocytes via distinct signaling pathways. *Osteoarthritis and Cartilage*. 2009;17(1):100-106. doi:10.1016/j.joca.2008.05.007

47. Rai MF, Graeve T, Twardziok S, Schmidt MFG. Evidence for Regulated Interleukin-4 Expression in Chondrocyte-Scaffolds under In Vitro Inflammatory Conditions. Zhou Z, ed. *PLoS ONE*. 2011;6(10):e25749. doi:10.1371/journal.pone.0025749

48. Porée B, Kypriotou M, Chadjichristos C, et al. Interleukin-6 (IL-6) and/or Soluble IL-6 Receptor Down-regulation of Human Type II Collagen Gene Expression in Articular Chondrocytes Requires a Decrease of Sp1·Sp3 Ratio and of the Binding Activity of Both Factors to the *COL2A1* Promoter. *Journal of Biological Chemistry*. 2008;283(8):4850-4865. doi:10.1074/jbc.M706387200

49. Daheshia M, Yao JQ. The interleukin 1β pathway in the pathogenesis of osteoarthritis. *Journal of Rheumatology*. 2008;35(12):2306-2312. doi:10.3899/jrheum.080346

50. *Principles of Osteoarthritis- Its Definition, Character, Derivation and Modality-Related Recognition*. InTech; 2012. doi:10.5772/1487

51. Meyaard L, Hovenkamp E, Keet IP, et al. Single cell analysis of IL-4 and IFN-gamma production by T cells from HIV-infected individuals: decreased IFN-gamma in the presence of preserved IL-4 production. *Journal of immunology (Baltimore, Md : 1950)*. 1996;157(6):2712-2718. http://www.ncbi.nlm.nih.gov/pubmed/8805678. Accessed March 23, 2020.

52. Lin T, Wang D, Nagpal ML, Chang W, Harrington Calkins J. Down-regulation of leydig cell insulin-like growth factor-i gene expression by interleukin-1. *Endocrinology*. 1992;130(3):1217-1224. doi:10.1210/endo.130.3.1537287

53. Rotwein P. Mapping the growth hormone-Stat5b-IGF-I transcriptional circuit. *Trends in Endocrinology and Metabolism*. 2012;23(4):186-193. doi:10.1016/j.tem.2012.01.001

54. Pichler J, Gerstmayr M, Szépfalusi Z, Urbanek R, Peterlik M, Willheim M. 1α,25(OH)2D3 Inhibits Not Only Th1 But Also Th2 Differentiation in Human Cord Blood T Cells. *Pediatric Research*. 2002;52(1):12-18. doi:10.1203/00006450-200207000-00005

55. Yoshikawa M, Nakajima T, Tsukidate T, et al. TNF-α and IL-4 regulate expression of IL-13 receptor α2 on human fibroblasts. *Biochemical and Biophysical Research Communications*. 2003;312(4):1248-1255. doi:10.1016/j.bbrc.2003.11.077

56. Sandoghchian Shotorbani S, Zhang Y, Baidoo SE, Xu H, Ahmadi M. IL-4 can inhibit IL-17 production in collagen induced arthritis. *Iranian journal of immunology : IJI*. 2011;8(4):209-217. doi:IJIv8i4A3

57. Cooney LA, Towery K, Endres J, Fox DA. Sensitivity and Resistance to Regulation by IL-4 during Th17 Maturation. *The Journal of Immunology*. 2011;187(9):4440-4450. doi:10.4049/jimmunol.1002860

58. Ye L, Wen Z, Li Y, et al. Interleukin-10 attenuation of collagen-induced arthritis is associated with suppression of interleukin-17 and retinoid-related orphan receptor γt production in macrophages and repression of classically activated macrophages. *Arthritis Research and Therapy*. 2014;16(2). doi:10.1186/ar4544

59. Newcomb DC, Zhou W, Moore ML, et al. A Functional IL-13 Receptor Is Expressed on Polarized Murine CD4 + Th17 Cells and IL-13 Signaling Attenuates Th17 Cytokine Production . *The Journal of Immunology*. 2009;182(9):5317-5321. doi:10.4049/jimmunol.0803868

60. Matsui K, Tsutsui H, Nakanishi K. Pathophysiological roles for IL-18 in inflammatory arthritis. *Expert Opinion on Therapeutic Targets*. 2003;7(6):701-724. doi:10.1517/14728222.7.6.701

61. PB | IL10-downregulated extracellular proteins [extracellular region]. https://reactome.org/PathwayBrowser/#/R-HSA-6783783&SEL=R-HSA-6784987. Accessed December 3, 2019.

62. Reactome | Expression of IL18, ALOX5. https://reactome.org/content/detail/R-HSA-6797293. Accessed November 14, 2019.

63. Honorati MC, Bovara M, Cattini L, Piacentini A, Facchini A. Contribution of interleukin 17 to human cartilage degradation and synovial inflammation in osteoarthritis. *Osteoarthritis and Cartilage*. 2002;10(10):799-807. doi:10.1053/joca.2002.0829

64. Goldring M. The role of the chondrocyte in osteoarthritis. *Arthritis & Rheumatism*. 1916;43(9):1916-1926. doi:10.1002/1529-0131(200009)43:9<1916::AID-ANR2>3.0.CO;2-I

65. PB | Expression of IL13-downregulated extracellular proteins. https://reactome.org/PathwayBrowser/#/R-HSA-6789325. Accessed November 14, 2019.

66. Zhou X, Spittau B, Krieglstein K. TGFβ signalling plays an important role in IL4-induced alternative activation of microglia. *Journal of Neuroinflammation*. 2012;9(1):706. doi:10.1186/1742-2094-9-210

67. Goldring MB, Otero M. Inflammation in osteoarthritis. *NIH Public access*. 2014;23(5):471-478. doi:10.1097/BOR.0b013e328349c2b1.Inflammation

68. Huey KA, McCusker RH, Kelley KW. Exaggerated expression of skeletal muscle-derived interleukin-6, but not TNFα, in mice lacking interleukin-10. *Journal of Neuroimmunology*. 2008;199(1-2):56-62. doi:10.1016/j.jneuroim.2008.05.004

69. PB | IL4,IL13-downregulated extracellular proteins [extracellular region]. https://reactome.org/PathwayBrowser/#/R-HSA-6785807&SEL=R-HSA-6789485&PATH=R-HSA-168256,R-HSA-1280215,R-HSA-449147. Accessed November 13, 2019.

70. Robinson K, Kenefeck R, Pidgeon EL, et al. Helicobacter pylori-induced peptic ulcer disease is associated with inadequate regulatory T cell responses. *Gut*. 2008;57(10):1375-1385. doi:10.1136/gut.2007.137539

71. Méndez-Samperio P, Miranda E, Vázquez A. Expression and Secretion of CXCL-8 and CXCL-10 From *Mycobacterium Bovis* BCG-Infected Human Epithelial Cells: Role of IL-4. *Mediators of Inflammation*. 2006;2006(1):1-6. doi:10.1155/MI/2006/67451

72. Aida Y, Maeno M, Suzuki N, Shiratsuchi H, Motohashi M, Matsumura H. The effect of IL-1β on the expression of matrix metalloproteinases and tissue inhibitors of matrix metalloproteinases in human chondrocytes. *Life Sciences*. 2005;77(25):3210-3221. doi:10.1016/j.lfs.2005.05.052

73. Cortez DM, Feldman MD, Mummidi S, et al. IL-17 stimulates MMP-1 expression in primary human cardiac fibroblasts via p38 MAPK- and ERK1/2-dependent C/EBP-β, NF-κB, and AP-1 activation. *American Journal of Physiology - Heart and Circulatory Physiology*. 2007;293(6). doi:10.1152/ajpheart.00928.2007

74. Zaragoza C, Balbín M, López-Otín C, Lamas S. Nitric oxide regulates matrix metalloprotease-13 expression and activity in endothelium. *Kidney International*. 2002;61(3):804-808. doi:10.1046/j.1523-1755.2002.00224.x

75. Blain EJ. Mechanical regulation of matrix metalloproteinases. *Frontiers in bioscience : a journal and virtual library*. 2007;12(February 2007):507-527. doi:10.2741/2078

76. Brown Lobbins ML, Shivakumar BR, Postlethwaite AE, Hasty KA. Chronic exposure of interleukin-13 suppress the induction of matrix metalloproteinase-1 by tumour necrosis factor α in normal and scleroderma dermal fibroblasts through protein kinase B/Akt. *Clinical and Experimental Immunology*. 2018;191(1):84-95. doi:10.1111/cei.13045

77. Forbes K, Webb MA, Sehgal I. Growth factor regulation of secreted matrix metalloproteinase and plasminogen activators in prostate cancer cells, normal prostate fibroblasts and normal osteoblasts. *Prostate Cancer and Prostatic Diseases*. 2003;6(2):148-153. doi:10.1038/sj.pcan.4500640

78. Li H, Wang D, Yuan Y, Min J. New insights on the MMP-13 regulatory network in the pathogenesis of early osteoarthritis. *Arthritis Research & Therapy*. 2017;19:248. doi:10.1186/s13075-017-1454-2

79. Merz D, Liu R, Johnson K, Terkeltaub R. IL-8/CXCL8 and Growth-Related Oncogene /CXCL1 Induce Chondrocyte Hypertrophic Differentiation. *The Journal of Immunology*. 2003;171(8):4406-4415. doi:10.4049/jimmunol.171.8.4406

80. Neidlin M, Chantzi E, Macheras G, Gustafsson MG, Alexopoulos LG. An ex vivo tissue model of cartilage degradation suggests that cartilage state can be determined from secreted key protein patterns. Sampen H-JI, ed. *PLOS ONE*. 2019;14(10):e0224231. doi:10.1371/journal.pone.0224231

81. Moriya C, Jinnin M, Yamane K, et al. Expression of matrix metalloproteinase-13 is controlled by IL-13 via PI3K/Akt3 and PKC- in normal human dermal fibroblasts. *Journal of Investigative Dermatology*. 2011;131(3):655-661. doi:10.1038/jid.2010.361

82. Wojdasiewicz P, Poniatowski AA, Szukiewicz D. The Role of Inflammatory and Anti-Inflammatory Cytokines in the Pathogenesis of Osteoarthritis. 2014. doi:10.1155/2014/561459

83. Sanchavanakit N, Saengtong W, Manokawinchoke J, Pavasant P. TNF-α stimulates MMP-3 production via PGE2 signalling through the NF-kB and p38 MAPK pathway in a murine cementoblast cell line. *Archives of Oral Biology*. 2015;60(7):1066-1074. doi:10.1016/j.archoralbio.2015.04.001

84. Hui W, Rowan AD, Cawston T. Insulin-like growth factor 1 blocks collagen release and down regulates matrix metalloproteinase-1, -3, -8, and -13 mRNA expression in bovine nasal cartilage stimulated with oncostatin M in combination with interleukin 1α. *Annals of the Rheumatic Diseases*. 2001;60(3):254-261. doi:10.1136/ard.60.3.254

85. Duivenvoorden WCM, Hirte HW, Singh G. Transforming growth factor β1 as an inducer of matrix metalloprotease expression and activity in human bone-metastasizing cancer

cells. *Clinical and Experimental Metastasis*. 1999;17(1):27-34. doi:10.1023/A:1026404227624

86. Chambers M, Kirkpatrick G, Evans M, Gorski G, Foster S, Borghaei RC. IL-4 inhibition of IL-1 induced Matrix Metalloproteinase-3 (MMP-3) expression in human fibroblasts involves decreased AP-1 activation via negative crosstalk involving of Jun N-terminal Kinase (JNK). *Experimental Cell Research*. 2013;319(10):1398-1408. doi:10.1016/j.yexcr.2013.04.010

87. Lau KS, Nakashima O, Aalund GR, et al. TNF-alpha and IFN-gamma induce expression of nitric oxide synthase in cultured rat medullary interstitial cells. *The American journal of physiology*. 1995;269(2 Pt 2):F212-7. doi:10.1152/ajprenal.1995.269.2.F212

88. Van Bezooijen RL, Papapoulos SE, Löwik CWGM. Effect of interleukin-17 on nitric oxide production and osteoclastic bone resorption: Is there dependency on nuclear factor-κB and receptor activator of nuclear factor κB (RANK)/RANK ligand signaling? *Bone*. 2001;28(4):378-386. doi:10.1016/S8756-3282(00)00457-9

89. Schuerwegh AJ, Dombrecht EJ, Stevens WJ, Van Offel JF, Bridts CH, De Clerck LS. Influence of pro-inflammatory (IL-1 alpha, IL-6, TNF-alpha, IFN-gamma) and anti-inflammatory (IL-4) cytokines on chondrocyte function. *Osteoarthritis and cartilage*. 2003;11(9):681-687. doi:10.1016/s1063-4584(03)00156-0

90. Yorimitsu M, Nishida K, Shimizu A, et al. Intra-articular injection of interleukin-4 decreases nitric oxide production by chondrocytes and ameliorates subsequent destruction of cartilage in instability-induced osteoarthritis in rat knee joints. *Osteoarthritis and Cartilage*. 2008;16(7):764-771. doi:10.1016/j.joca.2007.11.006

91. Yamane K, Ihn H, Asano Y, Jinnin M, Tamaki K. Antagonistic effects of TNF-alpha on TGF-beta signaling through down-regulation of TGF-beta receptor type II in human dermal fibroblasts. *Journal of immunology (Baltimore, Md : 1950)*. 2003;171(7):3855-3862. doi:10.4049/jimmunol.171.7.3855

92. Roman-Blas JA, Stokes DG, Jimenez SA. Modulation of TGF-b signaling by proinflammatory cytokines in articular chondrocytes. doi:10.1016/j.joca.2007.04.011

93. Krušlin B, Ulamec M, Tomas D. Prostate cancer stroma: An important factor in cancer growth and progression. *Bosnian Journal of Basic Medical Sciences*. 2015;15(2). doi:10.17305/bjbms.2015.449

94. Reactome | Platelet alpha granule contents [extracellular region]. https://reactome.org/content/detail/R-HSA-481033. Accessed November 13, 2019.

95. Kwak HJ, Park MJ, Cho H, et al. Transforming growth factor-β1 induces tissue inhibitor of metalloproteinase-1 expression via activation of extracellular signal-regulated kinase and Sp1 in human fibrosarcoma cells. *Molecular Cancer Research*. 2006;4(3):209-220. doi:10.1158/1541-7786.MCR-05-0140

96. Lee YJ, Lee EB, Kwon YE, et al. Effect of estrogen on the expression of matrix metalloproteinase (MMP)-1, MMP-3, and MMP-13 and tissue inhibitor of metalloproternase-1 in osteoarthritis chondrocytes. *Rheumatology International*. 2003;23(6):282-288. doi:10.1007/s00296-003-0312-5

97. Movahedi B, Van De Casteele M, Caluwé N, et al. Human pancreatic duct cells can produce tumour necrosis factor-α that damages neighbouring beta cells and activates dendritic cells. *Diabetologia*. 2004;47(6):998-1008. doi:10.1007/s00125-004-1426-3

98. PB | IL10-downregulated extracellular proteins [extracellular region]. https://reactome.org/PathwayBrowser/#/R-HSA-6783783&SEL=R-HSA-6784987. Accessed April 6, 2021.

99. Papaioannou AI, Kostikas K, Kollia P, Gourgoulianis KI. Clinical implications for vascular endothelial growth factor in the lung: Friend or foe? *Respiratory Research*. 2006;7. doi:10.1186/1465-9921-7-128
