# Supplementary material for: Regulatory network-based model to simulate the biochemical regulation of chondrocytes in healthy and osteoarthritic environments
Source: Sci Rep. 2022 Mar 9;12:3856. doi: 10.1038/s41598-022-07776-2 (PMC8907219; doi:10.1038/s41598-022-07776-2)
Supplement: Supplementary file 2 — Supplementary Information 2. [file 41598_2022_7776_MOESM2_ESM.pdf]

Regulatory network-based model to simulate the biochemical regulation of chondrocytes in healthy and osteoarthritic environments

Maria Segarra-Queralt, Michael Neidlin, Laura Tio, Jordi Monfort, Joan Carles Monllau, Miguel Á. González Ballester, Leonidas G. Alexopoulos, Gemma Piella, Jérôme Noailly

Additional Material 2. Fitness function exploration

We finned tunned the ENR network with a training set form Melas et *al.* (2014) with nine different fitness functions. Here can be seen 8, and the 9th is the one described in the manuscript.

$$MAD = \frac{\sum_{i=1}^{n} |\ln(X_i) - \ln(Y_i)|}{\text{n}} \quad (1)$$

$$MEAE = \frac{\sum_{i=1}^{n} e^{|X_i - Y_i|}}{\text{n}} \quad (2)$$

$$MAED = \frac{\sum_{i=1}^{n} \left|e^{(X_i)} - e^{(Y_i)}\right|}{\text{n}} \quad (3)$$

$$MRE = \frac{\sum_{i=1}^{n} \frac{|X_i - Y_i|}{Y_i}}{\text{n}} \quad (4)$$

$$MDRAE = \frac{\sum_{i=1}^{n} \frac{|X_i - Y_i|}{DR}}{\text{n}} \quad DR = 2 \quad (5)$$

$$MQD = \frac{\sum_{i=1}^{n} |(X_i - Y_i)^2}{\text{n}} \quad (6)$$

$$RMSE = \sqrt{\frac{\sum_{i=1}^{n} |(X_i - Y_i)^2}{\text{n}}} \quad (7)$$

$$ICOSIM = 1 - \frac{\sum_{i=1}^{n} X_i - Y_i}{\sqrt{\sum_{i=1}^{n} X_i^2}\sqrt{\sum_{i=1}^{n} Y_i^2}} \quad (8)$$

in each fitness function, X corresponds to the theoretical data and Y comes from experimental data. For each function we have done five rounds of optimisation with a genetic algorithm (we used MATLAB defaults options).We have selected the best network according to a qualitative evaluation and we have computed the numerical error (derived from the studied fitness function) and the relative error for the LIT, ENR and OPT network with the training and the independent validation data set.

However, we have done another version of each fitness function by adding a constrain regarding network baseline. This means that if the network baseline is catabolic, the fitness function adds an extra error to the natural error value. Then, we are forcing the optimization to explore only networks that have an anabolic nature. We have vectorized the genetic algorithm and we explore 200 networks for each objective function. Results can be seen separately by each fitness function in **A2 Table 1** and **A2 Table 2.**

A2 Table 1. No baseline constraint results. In parenthesis it can be seen the number of the network. BQN: Best quantitative network. BQL: Best qualitative network

**Fitness Function**		**NMAD%**		**Normalized Root Squared Error (% of > 0.15)**	**Qualitative evaluation**	
		Training	Indpendent Val		**Expected literature-based responses (+)%**	**Treatment (unexpected responses)**
**MEAE**	BQL (67)	15.2273	10.5433	19.4030	56.5789	↑ MMP14, PGE2 and TNFa.
	BQN (44)	11.2943	7.0917	12.0690	47.2973	↑ MMP13, 14, NO PGE2.
**MAED**	BQL (3)	9.8352	7.5811	13,4328	56.5789	↑ MMP13,MMP14.NO
	BQN (3)	“	“	“	“	“
**MRE**	BQL (24)	25.5117	10.1411	20,8955	77.0270	↑ VEGF, PGE2,MMP1,13 i 14
	BQN (8)	10.5684	11.6985	36.2069	57.3333	MMP1,13,MMP14 i VEGF
**MDRAE**	BQL (12)	7.8371	14.8386	37,3134	78.3784	OK (MMP14)
	BQN (12)	“	“	“	“	
**MQD**	BQL (2)	8.2764	4.2779	7,5	61.8421	↑MMPS VEGF
	BQN (28)	7.6983	9.0461	20,8955	54.6667	VEGF
**RMSE**	BQL (45)	7.9093	10.2313	25	66.2162	OK<br>↑MMPS 1, 13, 14
	BQN (13)	7.7302	7.9007	8,9552	64.8649	VEGF, PGE2,<br>MMP14,13,1
**ICosSim**	BQL (75)	17.7765	7.6055	19	72.9730	↑MMPS 1, 13, VEGF, CYCS and ADAMTs
	BQN (55)	12.7442	7.4881	10	39.4737	↑MMPS 14, VEGF, PGE2 and ADAMTs
**MAD**	BQL (13)	8.2269	7.5026	10,4478	71.8310	OK (MMP1)
	BQN (95)	8.1688	5.7143	“	55.4054	“

A2 Table 2. Baseline constraint results. ***BQN: Best quantitative network. BQL: Best qualitative network***

**Fitness Function**		**NMAD Error%**		**Normalized Root Squared Error (% of > 0.15)**	**Qualitative evaluation**	
		Training	Indpendent Val		**Qual Eval (+)%**	**Treatment (unexpected responses)**
**MEAE**	BQL (16)	7.9606	6.8367	13	75	↑VEGF↑PGE2 (↑MMP1,13,14)
	BQN (36)	7.6262	11.3886	"	50	"
**MAED**	BQL (71)	15.2273	10.5433	23	56.5789	OK(↑MMP3 &COL2A)
	BQN (3)	9.8352	7.5811	15	56.5789	↑VEGF(↑MMP3,13,1)
**MRE**	BQL(42)	27.9053	17.0929	70%	86.8421	↑MMPS VEGF, PGE2
	BQN (7)	11.1064	7.3834	"	50	"
**MDRAE**	BQL (98)	15.3168	12.2190	46.2687%	64.4737	OK
	BQN (54)	12.0134	12.2190	14,9253%	55.4054	↑VEGF,↑PGE2,↑NO (↑MMP1,13,14)
**MQD**	BQL (58)	12.2082	8.1218	24%	68.4211	OK (↑MMP13)
	BQN (74)	11.1327	13.3674	12%	67.1233	↑VEGF (↑MMP13)
**RMSE**	BQL (32)	12.8300	9.0295	22%	76.3158	OK (↑MMP13,3)
	BQN (45)	11.4349	11.0887	14,9253%	64.4737	OK (↑MMP1,3)
**AMD**	BQL (62)	13.0770	11.0381	32,8358	84.2105	OK(↑MMP14)
	BQN (73)	9.9251	7.6681	11,9403	55.4054	↑ NO(↑MMP1)
**ICosSim**	BQL ()	20.1863	12.6384	15	68.0556%	↑MMPS 1, 13, VEGF, CYCS and ADAMTs
	BQN ()	18.9123	6.6400	23	48.6111%	↑MMPS 14, VEGF, PGE2 and ADAMTs
**MAD**	BQL (29)	15.8005	14.3330	43.2836	71.0526	OK
	BQN (21)	10.7673	11.6401	"	59.2105	
