# Supplementary material for: Regulatory network-based model to simulate the biochemical regulation of chondrocytes in healthy and osteoarthritic environments
Source: Sci Rep. 2022 Mar 9;12:3856. doi: 10.1038/s41598-022-07776-2 (PMC8907219; doi:10.1038/s41598-022-07776-2)
Supplement: Supplementary file 3 — Supplementary Information 3. [file 41598_2022_7776_MOESM3_ESM.pdf]

Regulatory network-based model to simulate the biochemical regulation of chondrocytes in healthy and osteoarthritic environments

Maria Segarra-Queralt, Michael Neidlin, Laura Tio, Jordi Monfort, Joan Carles Monllau, Miguel Á. González Ballester, Leonidas G. Alexopoulos, Gemma Piella, Jérôme Noailly

Additional Material 3. Regulatory network with proper Extracellular-Matrix environments.

We have decided to add the following interaction in the optimized-based network based on 10.1053/joca.2002.0806:

1. COL2A activates TFG-b
2. ACAN activates FGF_2
3. COL2A activates ACAN.

Other interactions not reported in this document were excluded, as they were based on experiments performed with other cell types or tissues. We have included them directly in the optimized network, as a stimulation process on top of the experimentally calibrated and validated one (otherwise, one would expect that the optimization algorithm would remove these interactions, as changes in extracellular matrix cannot be reflected in the cell culture experiments of 25 min performed by Melas et al. 2014).

When we compare the results with the new interactions, error increases in every test of the quantitative validation. The cumulative error (computed with the Normalized Mean Absolute Error, NMAD) increases for both the training and validations sets to 14% and 12%, respectively (See Table 1 of this manuscript). Finally, when we plot the Normalized Root Squared Error (NRSE) error for each data point, the new added interactions decreases the responses that cannot be represented from 81% to 75% (see Fig. 1).

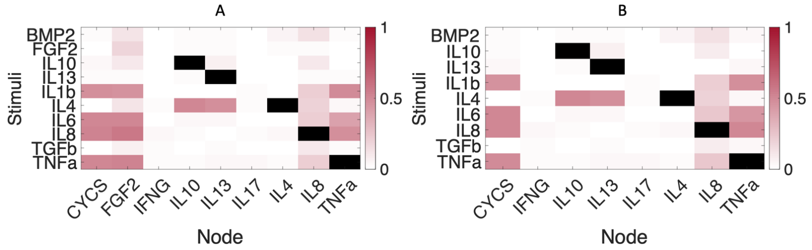

Fig. 1. Normalized Root Squared Error (NRSE) for the network with the interactions suggested by the reviewer (A) and the presented in the manuscript (B).

In contrast, the qualitative evaluation improves from 95% to 96%, meaning that the interactions are beneficial to the model in terms of interpretability. This suggests that when extracellular matrix interaction will be considered, for example in an agent-based modelling, when an extracellular matrix will be a potential regulator of the model (not the case of the experimental data), it will be useful to include them.

Table 1: Normalized mean absolute error NMAD as a representation of the cumulative error of each network regarding the training data from Melas et al. and validation data from Neidlin et al. The Extracellular Matrix (ECM) regulated network comes from adding the interactions mentioned in Response to question 21 to the optimized model.

**Network**	**Training (NMAD%)**	**Independent Validation (NMAD%)**
Optimized network	12.2096	7.9420
ECM network	14.1938	12.0030
